# Does context matter? A discrete choice experiment investigating the impact of palliative context on EQ-5D-5L health state valuation

**DOI:** 10.1007/s11136-025-04023-9

**Published:** 2025-07-29

**Authors:** Irina Kinchin, Peiwen Jiang, Deborah Street, Richard Norman, David Currow, Meera Agar, Charles Normand, Bridget Johnston, Peter May, Rosalie Viney, Brendan Mulhern

**Affiliations:** 1 Centre for Health Policy and Management, Trinity College Dublin, College Green, Dublin, 2 Ireland; 2 Faculty of Health, University of Technology Sydney, Sydney, NSW Australia; 3 School of Population Health, Curtin University, Perth, Australia; 4 Faculty of Science, Medicine and Health, University of Wollongong, Wollongong, NSW Australia; 5 Cicely Saunders Institute of Palliative Care, Policy and Rehabilitation, King’s College London, London, UK

**Keywords:** EQ-5D, Health state valuation, Discrete choice experiment, End of life, Palliative care

## Abstract

**Purpose:**

Ensuring the values applied in health technology assessment of palliative care accurately reflect the palliative context is crucial for informed and effective resource allocation. The aim of this study is to examine whether the valuation of EQ-5D health states varies when framed within a palliative care needs context: limited life expectancy and availability of supports.

**Methods:**

This study was a multinational cross-sectional discrete choice experiment (DCE) with respondents from the general populations of Australia (*n* = 2,082), Ireland (*n* = 1,280), and the UK (*n* = 2,009). Each participant was presented with a series of 20 choice sets, in which they were asked to choose between two EQ-5D-5L health states and immediate death. Half of the choice sets were accompanied by a “context vignette” while the remaining half were “context-free”. The context vignettes, developed through a four-stage iterative process, described four distinct levels of palliative care needs. A D-efficient DCE design was developed, and the data were analysed using multinomial logit regression models.

**Results:**

The study found inconsistencies in the EQ-5D-5L health state valuations in palliative contexts compared with context free valuation. Both life expectancy and level of support impacted health state valuation, with life expectancy having the larger effect. The inclusion of the palliative care vignettes substantially increased the number of health states that were given values worse than dead. This increase was more pronounced in Australia and the UK than in Ireland.

**Conclusion:**

These results imply that EQ-5D value sets that are context free require careful interpretation, especially when applied in settings such as palliative care.

**Supplementary Information:**

The online version contains supplementary material available at 10.1007/s11136-025-04023-9.

## Plain English summary

Why we did this study? Health technology assessments often use “quality-adjusted life years” (QALYs) to help decide which healthcare services to fund. QALYs are based on how the public values different health states, usually using generic values that do not consider specific situations like end-of-life care. We wanted to find out if adding a palliative care needs context—such as having a terminal illness with limited time and support—changes how people value health states, especially those considered “worse than dead,” in Australia, Ireland, and the UK.

How we did it? We surveyed 5,371 adults from Australia, Ireland, and the UK. Each participant completed two sets of choice tasks: one with no special context, and one with a short story (vignette) describing a palliative care needs context, with different life expectancies and support levels. In each task, participants chose the best and worst option from two health states and immediate death.

What we found: Adding a palliative care context changed how people valued health states. The proportion of health states considered “worse than dead” increased, especially in the scenario with only three months to live and limited support. For example, in Australia, this proportion rose from 15.5% (no context) to 32.5% (worst-case palliative context). Mobility was the most important health attribute across all countries. Australia showed the largest changes, while Ireland showed the smallest.

Why it matters? Using generic health state values may underestimate the value of palliative care. Our results suggest that economic evaluations for end-of-life care should consider context-specific values to better reflect societal preferences and support fair healthcare decisions.

## Introduction

In health technology assessment, health state valuation studies, which quantify individual preferences for health states are used to derive utilities for the estimation of Quality-Adjusted-Life-Years (QALYs) that inform the allocation of healthcare resources, treatment prioritisation, and development of health policies [1]. Discrete Choice Experiments (DCEs) are widely used in valuation studies, helping to identify the trade-offs individuals are willing to make among different health state attributes. These population preferences are then used to estimate value sets anchored on the full health – dead utility scale [2, 3].

The context-sensitive nature of health valuation, as emphasised by Schwappach [4] and Dolan [5], highlights the need for more refined valuation methods capable of capturing the nuances in diverse healthcare contexts. This particularly pertinent for palliative care, where the primary focus is on optimising function, or ensuring that the current state is at least maintained, and managing symptoms. Given the unique nature of this setting, evidence suggests that the context of palliative care needs might impact the valuation of health states, and the subsequent characteristics of the value sets produced [6, 7]. Until recently, the National Institute for Health and Care Excellence (NICE) recognised the unique nature of palliative care by employing an end-of-life premium. However, in a recent review, this premium was eliminated and replaced with a new “disease severity” modifier, indicating a shift in valuation methodologies [8]. 

In palliative care, both condition specific and generic instruments, including the EORTC-QLQ-C30, the McGill Quality of Life Questionnaire, the EQ-5D and ICECAP-SCM have been validated [9]. The EQ-5D is the most commonly used generic measure that allows both description and valuation of health-related quality of life for different health states, thus facilitating comparisons across various health conditions [10]. However, when used in palliative care, the values applied to EQ-5D health states may not reflect the trade-offs in relation to quality of life and length of life or across particular domains that are most pertinent in this setting. This limitation raises questions about the sensitivity of the EQ-5D value sets used, when applied to a palliative care context.

Moreover, research indicates significant disparities in the delivery of palliative care services internationally [11]. The human development index (measured by health, knowledge and standard of living) and the extent of palliative care development were negatively associated with the duration of palliative care. However, even among developed countries, such as Australia, Ireland, and the UK, differences in palliative care duration were observed. Specifically, Australia has a weighted median palliative care duration of just 6 days, while Ireland and the UK report significantly longer durations, at 46 and 48 days, respectively. These differences suggest that the fulfillment of palliative care needs and the corresponding quality of life values attributed to palliative care may vary considerably from one country to another.

This study aims to determine if preferences and resulting valuations for EQ-5D-5L health states, as when described within a palliative care needs context, differ from those derived without any context, in three English speaking countries, Australia, Ireland, and the UK. We developed vignettes to provide a description of a number of key features of a palliative care needs context [12–14]. Our investigation was guided by three research questions [RQs].

**RQ1:** Does the inclusion of a palliative care needs context change individuals’ valuation of health states as measured by the EQ-5D-5L?

**RQ2: **Does the inclusion of a palliative care context shift individuals’ preference for the dead health state?

**RQ3:** Are there differences in how the palliative care context affects values across the countries?

## Methods

### Overview of the experiment

Each participant undertook two DCEs (DCE 1 in a palliative care needs context and DCE 2 no context), with the order of presentation of DCE 1 and DCE 2 randomised across participants. Each DCE comprised 10 choice sets. In DCE 1, each choice set began with the presentation of a “palliative context needs vignette”, in which the respondent was asked to imagine they had a terminal illness with a specified prognosis or duration of life expectancy (3 months or 2 years) and available level of support (limited or adequate). This was followed by a choice set with three options: two EQ-5D-5L health states and immediate death. Participants were instructed to choose the most and the least preferred option in each choice set, given the context vignette. The context vignette was fixed for each respondent across all 10 choice sets but varied across respondents. Thus, DCE1 had four study arms which presented different context vignettes (Table 1).

**Table 1:** DCE arms used in the study

Study arm	Palliative context	
	Duration or prognosis	Support level
1	3-month life expectancy	Limited support
2	3-month life expectancy	Adequate support
3	2-year life expectancy	Limited support
4	2-year life expectancy	Adequate support

In DCE 2 there was no context vignette and respondents were presented with two EQ-5D-5L health states and immediate death and asked to choose the most and least preferred option. Appendix 1 in the supplementary materials provides examples of survey screenshots for both DCEs.

### Palliative context ‘vignette’ development

This study followed a four-step iterative process to develop and contextualise vignettes. The aim of *Stage 1* involved developing draft vignettes, based on a review of palliative care needs literature and quality of life instruments (for example, ASCOT, ICECAP-SCM). Key considerations included vignette format (story versus self-imagination), terminology (specific diseases versus generic terms), and content scope (symptoms only versus demographic characteristics). Vignettes were designed to be evidence-based while avoiding direct reference to EQ-5D-5L domains. *Stage 2* refined the draft vignettes through consultations with seven experts, including three palliative care clinicians, two health economists, and two patient representatives, to ensure clinical accuracy and study appropriateness. Consultations were conducted through a combination of one-to-one interviews and a group discussion, allowing for both individual and collective feedback on the vignettes. *Stage 3* involved pilot testing through six cognitive interviews with patient representatives from Voices4Care (UK and Ireland). Voices4Care is a public and patient involvement (PPI) group coordinated by the All Ireland Institute of Hospice and Palliative Care (AIIHPC). The group is composed of people with lived experience of palliative care, including patients, carers, and family members, as well as members of the public with an interest in palliative and end-of-life care. In Stage 3 of vignette development, members of Voices4Care were consulted to ensure that the vignettes were understandable, relevant, and sensitive to the experiences of people affected by palliative care. Subsequently, the vignettes were reviewed by Australian-based palliative care clinicians to ensure its relevance and accuracy for the local context. Based on feedback, vignettes were simplified: palliative duration was reduced to two options (3 months/2 years), ‘feeling prepared to die’ was removed, and ‘terminal illness’ replaced ‘life-threatening condition’ for clarity. *Stage 4* finalized the vignettes through ten cognitive interviews in Ireland and a pilot survey with 103 Australian respondents, ensuring comprehension and usability among the general population.

### The designed experiment

Each choice set consisted of two EQ-5D-5L states and immediate death. The approach to the construction of the health state pairs was to first construct all 3000 pairs of three attributes, each with five levels, with two dimensions better in option A than in option B and with the remaining dimension better in option B than in option A. From this set of 3000 pairs, 1000 random sets of 18 pairs were selected and the set of pairs with the highest efficiency, relative to the efficiency of the full set of 3000 pairs, was chosen. To construct the choice sets, we began with 18 pairs of options, each defined by three attributes with differing levels. Each of these pairs was then expanded into a set of 10 pairs, each involving five attributes. For each set, the original three attributes from the pair determined the levels for those attributes in both options A and B. The remaining two attributes were assigned the same level in both options, ensuring that only the attributes with differing levels contributed to the utility differences between options. This approach allowed us to systematically vary the attribute combinations while maintaining a manageable number of choice sets. While it does not matter at what levels the overlapped attributes are presented, in terms of design efficiency [15]. To ensure balanced representation of attribute levels, we used all 9 possible ordered pairs of the levels 2, 3, and 4 for the two overlapped attributes. Each ordered pair was used in two of the 18 choice sets, so that across the design, each combination was equally represented. The resulting set of choice sets was 92.1% efficient for non-informative priors, compared to the best possible when there must be overlap on two of the five dimensions, and where no dominated pairs are allowed. Finally, these 180 choice sets were grouped into blocks of 10 choice sets each, such that all 10 pairs of overlapped attributes appeared once each in each block and there were 10 different triples for the non-overlapped dimensions. The choice sets are available on request. We chose to overlap on two items as previous studies have shown that respondents find overlap on two dimensions less challenging than having no overlap [16].

While the typical guideline suggests having 20 respondents per choice set [17], empirical studies have shown that 80 respondents per choice set can reduce the standard error by about 50% [18]. Recent research has suggested having 50 respondents per choice set, or even 100 if confidence intervals for ratios are necessary [19]. Hence, for each country, we randomly allocated 100 respondents to each choice set. Within each arm, respondents were presented 20 choice sets in total, 10 choice sets with the palliative context vignette and 10 choice sets with no context vignette.

### Sample frame and recruitment

Respondents were sourced from the panel provider Pureprofile, which maintains representative panels of the general populations in Australia, the UK and Ireland by age groups and sex. All respondent data were anonymised.

Each respondent accessed and completed the survey at their convenience using a web link. The survey began with a series of baseline questions capturing information such as age, sex, partnership status, carer status, two warm-up DCE tasks, the 20 DCE choice sets, followed by additional demographic and socio-economic status questions, including country of birth and country where most time had been spent, religion, education (country specific), employment status, experience with a life-limiting illness, and experience caring for someone who has passed away, experience with severe health problems, ethnic background, whether a language other than English was spoken at home, self-rated health status using EQ-5D-5L, and feedback questions.

### Statistical analysis

#### Descriptive analysis

Descriptive statistics compared the health and socio-demographic characteristics of the participants across Australia, the UK and Ireland including age, sex, marital status, experience providing care to someone, whether they had children, religion, engagement in religious activities, employment status, education, experience with a life-limiting illness, health problems, ethnicity, language, and quality of life.

#### Modelling choice data

The choice data were analysed under the framework of random utility theory, assuming that respondents make choices that maximise their utility. Since the aim of this study was to compare the overall magnitude of the dimensions rather than assessing the preference heterogeneity, for this paper the multinomial logit model (MNL) was employed to analyse the choice data. The MNL assumes individuals within the sample population have uniform preferences.

First, MNL models with interactions were fitted to assess whether ‘context’ had impacts on the preferences for EQ-5D-5L dimensions:

- Shorter or longer life expectancies, using a 3-level categorical variable representing no context, a 3-month life expectancy, and a 2-year life expectancy.
- Presence and quality of support, using a 3-level categorical variable for no context, limited support, and adequate support.
- Combinations of life expectancy and support levels.
- Inclusion or exclusion of contextual details, such as with or without additional context.

Separate models were then fitted for each country and each study arm to examine how preferences for EQ-5D-5L dimensions differed ‘in context’ across the three countries. In order to compare estimates across various subgroups, the coefficients were anchored on the full health-dead scale. The normalisation process involved dividing the coefficients corresponding to each level of each dimension by the value of the dead variable.

Relative attribute importance (RAI) scores were calculated for palliative context and no context scenarios across each country and each study arm. The RAI scores were calculated by dividing the coefficient for level 5 (severe problems) in each dimension of the EQ-5D-5L by the sum of coefficients for level 5 for all five dimensions, as shown in Eq. (1).

1 $${RAI}_{k5}=\frac{{\beta }_{k5}}{{\beta }_{MO5}+{\beta }_{SC5}+{\beta }_{UA5}+{\beta }_{PD5}+{\beta }_{AD5}}$$

where ${\beta }_{k5}$ is a coefficient of the worst level for the EQ-5D-5L dimension k.

All statistical modelling and analyses were performed using R [20]/Rstudio [21] with MNL models estimated using the *mlogit* package [22].

### Ethics

This study has been approved by the ethics committees at Trinity College Dublin [03E/2021/07 granted on 07 February 2022], Curtin University [HRE2022-0096 granted on 23 February 2022], University of Technology Sydney [HREC REF NO. ETH22-7049 granted on 12 April 2022], and King’s College London [LRS-21/22-29,058 granted on 02 September 2022].

## Results

### Sample characteristics

From the total number of 6,286 individuals who began the survey, 915 did not complete it, resulting in 5,371 completed responses. This group consisted of 2,082 participants from Australia, 1,280 from Ireland, and 2,009 from the UK. The median completion time for this survey was 14.0 min in Australia, 11.2 min in Ireland and 13.0 min in the UK. For our analysis, we used data from the 5,371 respondents who completed the full survey as there was no significant difference in MNL estimates before and after excluding respondents who spent less than 40% of the median completion time and consistently selected the same response for all questions (Appendix 2, Table 1 in supplementary materials; all models included in the sensitivity analysis are available upon request).

Table 2 provides the demographic characteristics of the analysed respondents from each country. Notable demographic differences included Ireland’s younger, more employed population compared to Australia and UK’s older, more retired respondents. Irish respondents also showed higher rates of religious involvement, multilingualism, and better self-reported health (except anxiety/depression). UK and Australian respondents had lower educational attainment and reported more health issues, though UK participants reported less anxiety/depression (Table 2).

**Table 2:** Socio-demographic characteristics of respondents from each country

	AU *n(%)*	IE *n(%)*	UK *n(%)*
Age (study sample)			
18–24	240 (11.5)	252 (19.7)	197 (9.8)
25–34	412 (19.8)	345 (27)	322 (16.0)
35–44	367 (17.6)	255 (19.9)	322 (16.0)
45–54	337 (16.2)	225 (17.6)	376 (18.7)
55–64	331 (15.9)	118 (9.2)	358 (17.8)
65–74	249 (12)	75 (5.9)	269 (13.4)
75–84	133 (6.4)	10 (0.8)	157 (7.8)
85 and over	13 (0.6)	0 (0.0)	8 (0.4)
Age (national)*	[29]	[30]	[31]
18–24	6.3%	8.2%	8.3%
25–34	14.3%	13.8%	13.5%
35–44	13.9%	15.7%	13.0%
45–54	12.6%	13.1%	13.3%
55–64	11.6%	10.7%	12.6%
65–74	9.4%	7.8%	9.9%
75–84	5.6%	4.1%	6.1%
85 and over	2.1%	1.4%	2.4%
Female	1073 (51.5)	647 (50.5)	1000 (49.8)
Married/ have partner	1245 (59.8)	781 (61.0)	1208 (60.1)
Never provided care before	1094 (52.5)	631 (49.3)	1,239 (61.7)
Have dependent children	816 (39.2)	564 (44.1)	643 (32.0)
Religion–not at all important	917 (44)	373 (29.1)	973 (48.4)
Never engage in religious activities	1095 (52.6)	357 (27.9)	1147 (57.1)
Employed	1267 (60.9)	899 (70.2)	1144 (56.9)
Hold university degree or higher	809 (38.9)	557 (43.5)	763 (38.0)
Experience of someone developing a life-limiting illness	1268 (60.9)	852 (66.6)	1199 (59.7)
Experience caring for someone with a life-limiting illness	932 (44.8)	620 (48.4)	838 (41.7)
Experience of someone dying from a life-limiting illness	711 (34.1)	471 (36.8)	653 (32.5)
Currently have severe health problems	205 (9.8)	111 (8.7)	211 (10.5)
Severe health problems in the past	238 (11.4)	171 (13.4)	209 (10.4)
Someone in the family has or had severe health problems	909 (43.7)	554 (43.3)	800 (39.8)
Currently caring for someone with health problems	239 (11.5)	127 (9.9)	230 (11.4)
Work in the healthcare sector	138 (6.6)	129 (10.1)	131 (6.5)
Ethnicity: white	1667 (80.1)	1121 (87.6)	1732 (86.2)
Speak English at home	1724 (82.8)	827 (64.6)	1604 (79.8)
Mobility (No problem)	1581 (75.9)	1044 (81.6)	1471 (73.2)
Self-care (No problem)	1870 (89.8)	1120 (87.5)	1697 (84.5)
Usual activities (No problem)	1604 (77.0)	1028 (80.3)	1461 (72.7)
Pain/discomfort (No problem)	1077 (51.7)	745 (58.2)	1063 (52.9)
Anxiety/depression (No problem)	985 (47.3)	601 (47)	1094 (54.5)
Median self-reported health rating (0–100)	76	76	75.5

Comparison with national statistics showed a higher proportion of younger adults (aged 18–34) and a lower proportion of older adults (aged 75 and above) in the study sample compared to the general populations in Australia, Ireland, and the UK. The gender distribution was similar to national figures, with approximately half of respondents identifying as female in each country. Other characteristics, such as marital status, employment, and education, were broadly comparable to national profiles, though the proportion with a university degree was somewhat higher in the sample.

### The impact of context on the EQ-5D-5L health states values

#### Results from MNL models with interactions

Appendix 3 in the supplementary materials detail results from a range of MNL models with interaction terms which were estimated to examine how life expectancy (3-month versus 2-year), support level (limited and adequate), and varying combinations of life expectancy and support levels, affected health state values. Overall, the available level of support and the duration of life expectancy had a consistent statistically significant influence only on the death attribute. Although there were some significant interactions between the EQ-5D-5L coefficients, no consistent pattern was observed.

Appendix 3 Tables 1a-c show the impact of life expectancy on the valuation of health states. The results suggest that the influence of life expectancy was most pronounced in the UK sample compared to those in Australia and Ireland. In the UK, both 3-month and 2-year life expectancy scenarios had a significant impact on the death coefficient.

Appendix 3 Tables 2a-c present the impact of support level on health state values. The interaction between limited support and the death attribute was statistically significant across Australia, the UK and Ireland, whereas the interaction with adequate support was not significant.

Subsequently, the impact of each of the four contexts was examined individually, as documented in Appendix 3 Tables 3a-c. Table 3a findings show that in the Australian sample, only the worst-case scenario—3-month life expectancy with limited support—significantly impacted the death attribute, indicating a critical shift in death perception under these conditions. The other three vignettes did not show significant effects. Table 3b shows that only the interaction between a 2-year life expectancy with limited support context and the death attribute was statistically significant in the Irish sample. Spending two years in an environment with insufficient support may be perceived as prolonged suffering, making death seem preferable to continued life under such conditions. As shown in Table 3c, among the UK respondents, the 3-month life expectancy with limited support vignette was also significant. Additionally, the interaction term between context and no context in the 3-month life expectancy with adequate support vignette was significant. This indicates that the duration of life expectancy plays a crucial role in shaping how death is valued in the UK sample.

Finally, Table 3 provides results for each study arm to investigate the impact of contextual vignette information on health state valuation in the Irish sample (Australian and UK data presented in Appendix 3, Tables 4a-b the supplementary materials). The interaction analysis between health states and contextual scenarios revealed minimal systematic effects. Notable significant interactions included death health state in study arm 1 for Australia (0.483, p = 0.006), mobility levels MO2 and MO3 in study arms 3 and 4 respectively for Ireland (0.237, p = 0.032; −0.262, p = 0.007), and MO5 in study arm 2 for the UK (0.429, p = 0.013). While the magnitude of coefficients varied across countries, the pattern of effects remained relatively consistent. Model fit statistics indicated better performance in study arms 3 and 4 across all countries (Log-likelihood ranges: UK: -8616 to -9288; Australian: -9488 to -10,285; Irish: -5846 to -6154).

**Table 3:** Separate MNLs with main effects and interactions between a dummy indicating a vignette with or without a context and each attribute level for each study arm in the Irish sample

	Study Arm 1			Study Arm 2			Study Arm 3				Study Arm 4		
	Coef	SE	P	Coef	SE	P	Coef	SE	P	Coef		SE	P
MO2	−0.196	0.078	**0.012**	−0.204	0.078	**0.009**	−0.413	0.079	** < 0.001**	−0.135		0.077	0.079
MO3	−0.307	0.069	** < 0.001**	−0.323	0.070	** < 0.001**	−0.246	0.070	** < 0.001**	−0.148		0.068	**0.029**
MO4	0.361	0.098	** < 0.001**	0.131	0.099	0.188	0.192	0.097	**0.049**	−0.141		0.101	0.162
MO5	−0.821	0.149	** < 0.001**	−0.609	0.147	** < 0.001**	−0.833	0.143	** < 0.001**	−0.631		0.146	** < 0.001**
SC2	−0.154	0.094	0.101	−0.236	0.096	**0.014**	−0.145	0.095	0.128	0.149		0.096	0.122
SC3	−0.012	0.089	0.893	0.093	0.092	0.310	−0.044	0.089	0.622	−0.007		0.089	0.935
SC4	−0.389	0.093	** < 0.001**	−0.529	0.096	** < 0.001**	−0.390	0.095	** < 0.001**	−0.425		0.093	** < 0.001**
SC5	−0.037	0.109	0.738	0.101	0.109	0.353	−0.139	0.112	0.212	−0.086		0.106	0.415
UA2	−0.119	0.095	0.207	−0.059	0.095	0.537	−0.152	0.097	0.116	−0.122		0.093	0.193
UA3	0.138	0.086	0.107	0.154	0.085	0.070	0.149	0.086	0.084	0.409		0.083	** < 0.001**
UA4	−0.367	0.097	** < 0.001**	−0.336	0.101	**0.001**	−0.312	0.099	**0.002**	−0.627		0.098	** < 0.001**
UA5	−0.323	0.104	**0.002**	−0.105	0.105	0.316	−0.187	0.104	0.071	−0.198		0.107	0.065
PD2	−0.160	0.094	0.088	−0.105	0.095	0.267	−0.105	0.096	0.272	−0.250		0.094	**0.008**
PD3	0.189	0.086	**0.029**	0.278	0.086	**0.001**	0.227	0.088	**0.010**	0.130		0.086	0.132
PD4	−0.526	0.100	** < 0.001**	−0.519	0.101	** < 0.001**	−0.631	0.102	** < 0.001**	−0.522		0.102	** < 0.001**
PD5	−0.226	0.101	**0.025**	−0.328	0.101	**0.001**	−0.341	0.102	**0.001**	−0.226		0.099	**0.023**
AD2	−0.166	0.096	0.082	−0.063	0.097	0.515	−0.255	0.097	**0.009**	−0.222		0.096	**0.020**
AD3	−0.020	0.093	0.832	0.204	0.094	**0.030**	0.181	0.094	0.054	0.152		0.093	0.104
AD4	−0.466	0.100	** < 0.001**	−0.550	0.104	** < 0.001**	−0.365	0.102	** < 0.001**	−0.450		0.099	** < 0.001**
AD5	−0.188	0.096	0.051	−0.141	0.096	0.142	−0.314	0.098	**0.001**	−0.349		0.095	** < 0.001**
Death	−2.577	0.161	** < 0.001**	−2.563	0.167	** < 0.001**	−2.801	0.166	** < 0.001**	−2.343		0.161	** < 0.001**
Interaction terms													
MO2	−0.011	0.110	0.921	−0.032	0.110	0.774	0.237	0.110	**0.032**	0.024		0.109	0.828
MO3	0.030	0.097	0.757	0.041	0.099	0.678	0.020	0.099	0.843	−0.262		0.096	**0.007**
MO4	−0.168	0.139	0.227	−0.022	0.141	0.873	−0.158	0.138	0.252	0.068		0.143	0.632
MO5	−0.090	0.214	0.672	−0.111	0.210	0.596	0.131	0.201	0.515	0.168		0.205	0.413
SC2	−0.067	0.132	0.614	−0.052	0.135	0.702	−0.106	0.134	0.430	−0.191		0.136	0.162
SC3	−0.034	0.125	0.786	0.060	0.130	0.645	0.169	0.125	0.178	0.057		0.127	0.652
SC4	0.034	0.130	0.792	0.068	0.134	0.615	−0.080	0.133	0.547	−0.248		0.131	0.059
SC5	0.042	0.154	0.786	−0.011	0.153	0.942	−0.138	0.158	0.383	0.072		0.152	0.633
UA2	0.075	0.133	0.575	−0.103	0.134	0.439	−0.057	0.136	0.677	−0.094		0.132	0.476
UA3	0.120	0.120	0.320	0.071	0.120	0.555	0.025	0.121	0.834	−0.197		0.117	0.093
UA4	−0.074	0.137	0.587	−0.027	0.143	0.848	−0.086	0.139	0.537	0.190		0.139	0.172
UA5	0.044	0.147	0.766	−0.013	0.148	0.928	0.039	0.147	0.792	−0.040		0.152	0.792
PD2	0.065	0.132	0.625	0.053	0.134	0.691	−0.015	0.134	0.911	0.198		0.133	0.138
PD3	−0.006	0.121	0.960	−0.010	0.121	0.932	−0.108	0.123	0.382	−0.045		0.123	0.715
PD4	0.013	0.141	0.928	0.042	0.143	0.768	0.056	0.144	0.697	−0.007		0.145	0.960
PD5	−0.030	0.143	0.834	−0.167	0.144	0.247	0.116	0.144	0.419	−0.066		0.141	0.642
AD2	−0.112	0.135	0.407	−0.234	0.137	0.088	0.247	0.138	0.073	0.149		0.136	0.271
AD3	0.164	0.131	0.211	0.005	0.132	0.969	−0.074	0.132	0.577	−0.154		0.132	0.245
AD4	0.035	0.141	0.806	0.150	0.146	0.306	0.132	0.143	0.357	0.063		0.140	0.656
AD5	0.092	0.135	0.496	−0.015	0.136	0.910	−0.098	0.138	0.475	−0.112		0.136	0.409
Death	0.411	0.226	0.069	0.081	0.234	0.730	0.489	0.232	**0.035**	−0.199		0.229	0.384
LL			−6154			−5846			−5928				−5999
AIC			12,392			11,776			11,940				12,082
BIC			12,676			12,059			12,224				12,366

Significant terms are bolded; Coef coefficient estimate, *SE* standard error; *LL* log-likelihood, *AIC* Akaike’s information criteria; *BIC* Bayesian information criteria; Study arm 1, a 3-month life expectancy with limited support vignette; Study arm 2, a 3-month life expectancy with adequate support vignette; Study arm 3, a 2-year life expectancy with limited support vignette; Study arm 4, a 2-year life expectancy with adequate support vignette

#### Results from separate MNL models

Separate MNL models^1^ were fitted for the context DCE and the no context DCE across each of the four study arms (defined by the context vignette) across each country. The magnitude of the coefficients within each dimension should increase reflecting greater deviations from the baseline as the severity of levels increases. Most of the coefficients were ordered as expected. To address nonmonotonicity in the dimension levels, we examined the coefficient estimates. Some levels showed unexpected patterns (either reversed ordering or inconsistent magnitudes compared to what theory would predict). To improve interpretability while maintaining model validity, we constrained these nonmonotonic coefficients to have the same value, effectively combining levels to share the same decrement from the baseline (i.e. no problem).

Table 4 presents Irish data, with Australian and the UK data provided in the supplementary materials (Appendix 2, Tables 2a-c). The comparison between context and no-context scenarios shows mixed results across different dimensions and study arms. In study arm 1 (3-month life expectancy with limited support), while some dimensions like mobility and self-care showed larger decrements in the context scenario (for example, MO5: -0.555 vs -0.374; SC4: -0.287 vs -0.215), this pattern was not consistent across all dimensions. For instance, in usual activities, the context decrements were actually smaller or similar to no-context decrements (UA4: -0.106 vs -0.135; UA5: -0.235 vs -0.261), and in anxiety/depression, the extreme level showed similar magnitude but slightly smaller decrement in context (AD5: -0.305 vs -0.326). Across study arms 2–4, the patterns remained variable, with differences between context and no-context scenarios showing no consistent direction or magnitude. For example, in study arm 3, some decrements were larger in the context scenario (SC5: -0.378 vs -0.256), while others showed the opposite pattern (MO2: -0.076 vs -0.147). The variability in the data suggests that the influence of palliative care context on health state valuations appears to be less systematic than might have been hypothesized.

**Table 4:** Anchored and adjusted EQ-5D-5L decrements in the Irish sample

	Study arm 1		Study arm 2		Study arm 3		Study arm 4	
	Context	No context	Context	No context	Context	No context	Context	No context
Mobility								
MO2	−0.095	−0.076	−0.095	−0.080	−0.076	−0.147	−0.044	−0.058
MO3	−**0.179**	−**0.125**	−**0.187**	−**0.181**	−**0.168**	−**0.201**	−0.205	−0.121
MO4	−**0.179**	−**0.125**	−**0.187**	−**0.181**	−**0.168**	−**0.201**	−0.233	−0.181
MO5	−0.555	−0.374	−0.455	−0.392	−0.463	−0.464	−0.415	−0.450
Self-care								
SC2	−0.102	−0.060	−0.116	−0.092	−**0.082**	−0.052	−**0.007**	**0.000**
SC3	−0.123	−0.064	−**0.147**	−**0.159**	−**0.082**	−0.067	−**0.007**	**0.000**
SC4	−**0.286**	−0.215	−**0.147**	−**0.159**	−0.258	−0.207	−0.261	–0.121
SC5	−**0.286**	−0.229	−0.204	−0.222	−0.378	−0.256	−0.267	–0.158
Usual activities								
UA2	**0.000**	−**0.020**	−**0.020**	−0.023	−0.090	−**0.028**	−**0.085**	**0.000**
UA3	**0.000**	−**0.020**	−**0.020**	−**0.029**	−0.015	−**0.028**	−**0.085**	**0.000**
UA4	−0.106	−0.135	−0.121	−**0.029**	−0.187	−0.113	−0.174	–0.145
UA5	−0.235	−0.261	−0.169	−0.135	−0.251	−0.179	−0.267	−0.230
Pain/discomfort								
PD2	−**0.002**	**-0.026**	**0.000**	**0.000**	−**0.026**	−0.037	−0.020	−**0.079**
PD3	−**0.002**	**-0.026**	**0.000**	**0.000**	−**0.026**	−**0.070**	−**0.091**	−**0.079**
PD4	−0.197	−0.193	−0.105	−0.135	−0.249	−**0.070**	−**0.091**	−0.274
PD5	−0.315	−0.281	−0.305	−0.263	−0.346	−0.304	−0.310	−0.370
Anxiety/depression								
AD2	−**0.095**	−**0.069**	−**0.078**	−0.025	−0.004	−**0.059**	−**0.030**	−**0.063**
AD3	−**0.095**	−**0.069**	−**0.078**	−**0.053**	−**0.008**	−**0.059**	−**0.030**	−**0.063**
AD4	−0.261	−0.253	−0.197	−**0.053**	−**0.008**	−0.156	−0.182	−0.222
AD5	−0.305	−0.326	−0.260	−0.215	−0.236	−0.268	−0.363	−0.371

Study arm 1, Context: a 3-month life expectancy with limited support; Study arm 2, Context: a 3-month life expectancy with adequate support; Study arm 3, Context: a 2-year life expectancy with limited support; Study arm 4, Context: a 2-year life expectancy with adequate support. The attribute levels in bold were constrained to be the same

The Australian data (Appendix 2, Table 3a) shows similar variability but with generally larger magnitude coefficients. For instance, in study arm 1, mobility level 5 shows stronger context effects (MO5: -0.606 vs -0.467) compared to the Irish data. The UK data (Appendix 2, Table 3c) also demonstrates mixed patterns. For example, in study arm 1, mobility level 5 shows similar context effects to Australia (MO5: -0.607 vs -0.532), but other dimensions show more modest differences between context and no-context scenarios.

Across all three countries, the impact of palliative care context on health state valuations showed variable patterns across different scenarios, with no clear evidence that the worst-case scenario (study arm 1) consistently produced larger decrements compared to milder scenarios. The influence of context appeared to be more complex and less dependent on scenario severity.

**Fig. 1:**
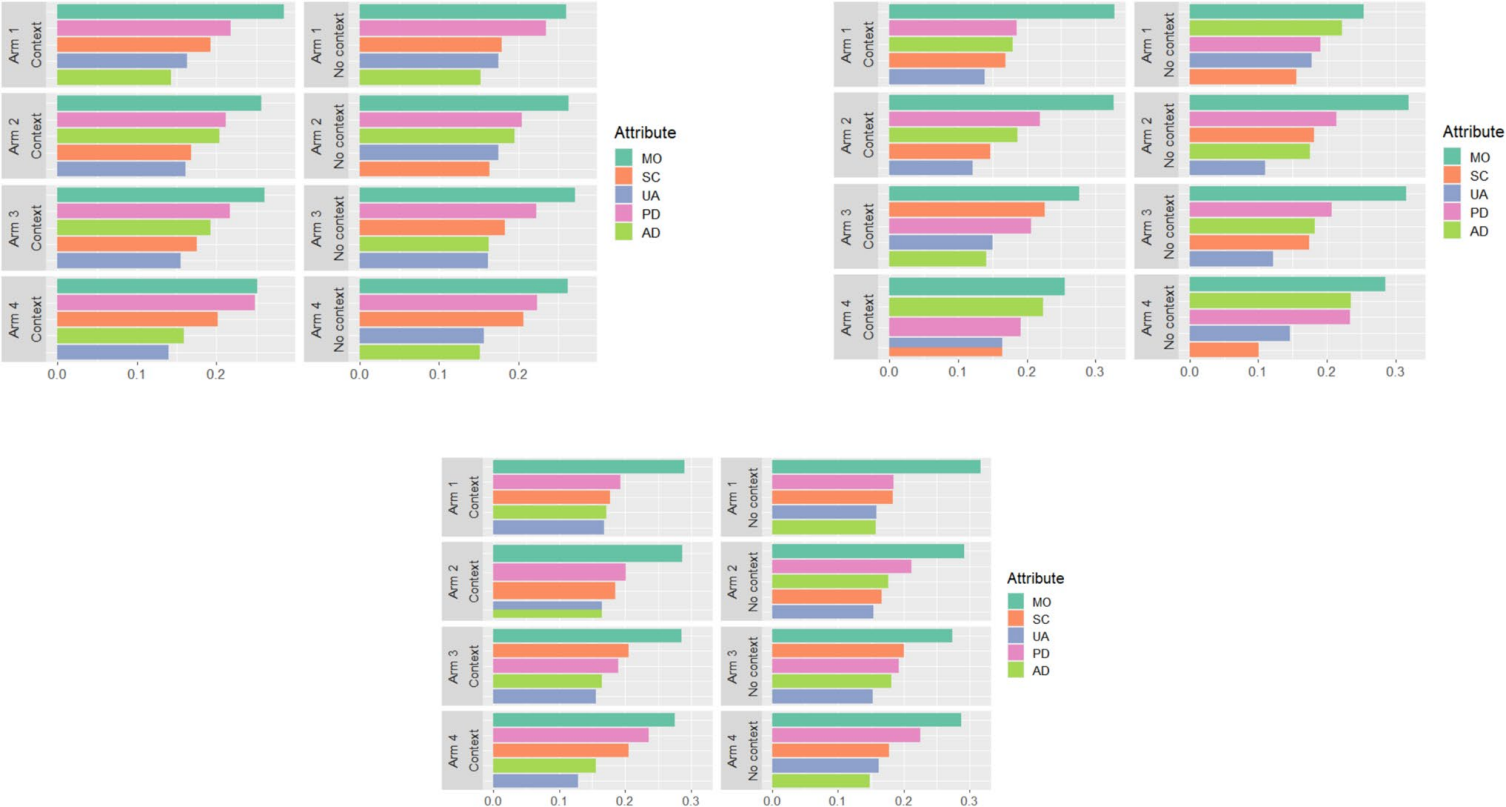
Relative attribute importance in three countries. **a** Relative attribute importance in the Australian sample. **b** Relative attribute importance in the Irish sample.*: Arm* 1*, Context*: a 3-month life expectancy with limited support; *Arm 2, Context*: a 3-month life expectancy with adequate support; *Arm* 3*, Context*: a 2-year life expectancy with limited support; *Arm* 4*, Context*: a 2-year life expectancy with adequate support

Figure 1 illustrates the relative importance scores across Australia, Ireland, and the UK for the four study arms. Mobility consistently emerged as the most important attribute in all three countries, regardless of variations in the contexts. In Irish Arm 1, one interesting observation is that the importance of mobility was comparatively lower under the no context scenario, compared to the worst-case scenario (i.e. a 3-month prognosis and limited support). In addition, preference heterogeneity was observed, as indicated by the variations in RAI scores under the no context scenarios among different study arms. However, when comparing RAI scores between palliative care context and no context scenarios, only minor differences were observed across four study arms and three countries.

### The impact of context on preferences for the “death” health state

**Fig. 2:**
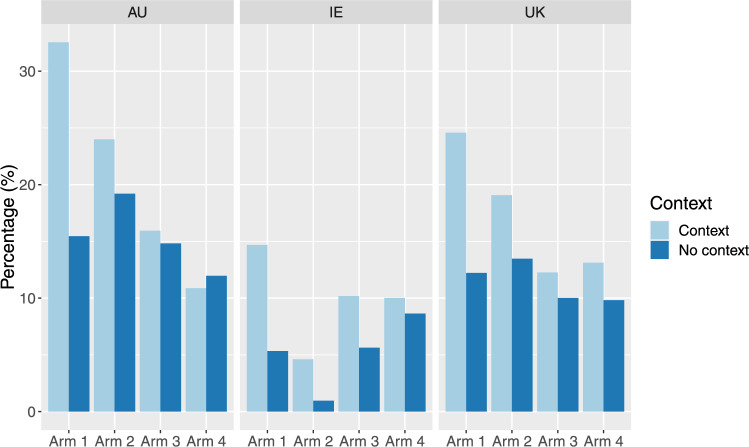
Influence of palliative care needs context on EQ-5D-5L health states considered worse than death by country

Overall, the number of health states worse than dead was largest in the Australian sample, while the Irish sample had the lowest number of such states (Fig. 2). In addition, the impact of palliative care contexts was evident in the percentages of health states worse than dead, with the most significant effect in the worst-case scenario (arm 1: the palliative care context with a 3-month life expectancy with limited support). Specifically, under the no context scenario, there were 15.5%, 5,3% and 12.2% health states considered to be worse than dead in Australia, Ireland, and the UK respectively. These percentages increased when respondents were presented with the palliative care scenario (arm 1: a 3-month life expectancy with limited support), with 32.5%, 14.7% and 24.6% in Australia, Ireland, and the UK respectively.

## Discussion

This study provides the first empirical evidence of how palliative care contexts influence EQ-5D-5L health state valuations across three countries, Australia, Ireland, and the UK. Our findings have implications for health economic evaluation and resource allocation in palliative care. The increased proportion of health states deemed "worse than dead" under palliative contexts—particularly with shorter life expectancy (3 months vs. 2 years)—suggests that societal preferences for end-of-life health states differ from those in non-palliative scenarios. This challenges the assumption that generic population value sets are universally applicable, especially for interventions targeting severe or life-limiting conditions.

### Policy and decision-making relevance

These findings inform how cost-effectiveness analyses (CEAs) for palliative care interventions should be conducted. For example, using generic value sets in CEAs may underestimate the perceived value of palliative interventions that extend life by even a few months, as respondents placed greater weight on preserving life when less time remained. Policymakers and agencies like NICE could use this evidence to advocate for context-specific modifiers in economic evaluations, ensuring that palliative care interventions—which often focus on quality rather than quantity of life—are assessed using values that reflect societal priorities in end-of-life scenarios. This would align with NICE’s recent emphasis on prioritizing health benefits in severe diseases [8] and could prevent undervaluation of palliative care in funding decisions.

The cross-country variations in valuations (e.g., Ireland’s lower proportion of "worse than dead" states) further highlight the need for localized or subgroup-specific value sets. Demographic factors such as religiosity, caregiving experience, and age—all of which differed across samples—likely shape societal preferences. For instance, Ireland’s younger, more religious sample may reflect cultural attitudes toward end-of-life care that differ from Australia or the UK. Policymakers could use these insights to tailor palliative care resource allocation to populations with distinct demographic profiles, ensuring equity in access and acceptability of services.

### Improving patient outcomes and resource efficiency

By quantifying how context alters health state valuations, this study provides insights for designing patient-centred palliative care models. For example, the strong preference against states perceived as "worse than dead" in short life expectancy scenarios suggests that patients may prioritize interventions minimizing suffering over life extension—a finding that could guide clinical guidelines and advance care planning. Similarly, health systems could use these results to prioritize funding for interventions that align with societal values (e.g., home-based palliative care over aggressive, costly end-of-life hospitalizations).

This study provides the first empirical evidence of the impact of a palliative context on EQ-5D-5L health state values across Australia, Ireland, and the UK. The introduction of a palliative care context increased the number of health states given values worse than dead, across all three countries. The impact was particularly prominent in palliative care contexts with a shorter life expectancy of 3 months, compared to those with a life expectancy of 2 years. This implies that respondents may value health differently in a setting in which they know that death is imminent. One way of interpreting this is that people may value life over death more when the available amount of life is more. This is an idea that has been suggested previously in theoretical and conceptual research [23, 24], but this has not been demonstrated widely in empirical data. It is a crucial point for health state valuation methods to consider, particularly in the context of suggestions that general population values can be used to assess states worse than death in economic evaluation [25].

While the overall impact of a palliative care context on the EQ-5D-5L health state valuation remained consistent across the three countries, variations in the percentages of health states worse than dead were observed. Interestingly, the Irish sample showed the smallest proportion of health states worse than dead among the 3125 health states, irrespective of palliative care needs. This discrepancy may be attributed to dissimilarities in demographic characteristics across the three countries. Specifically, Ireland had a higher percentage of younger and employed respondents, as well as a greater proportion of individuals reporting having adult children, placing importance on religion, and engaging in religious activities to some extent, in comparison to respondents from the UK and Australia. In line with previous research [26–28], demographic factors, including age, mental health status, employment status, and caregiving experience, may influence preferences regarding the death health state. It is especially relevant when assessing the cost-effectiveness of interventions aimed at specific demographic groups.

### Strength and limitations

The strengths of this study lie in its comprehensive approach to examining the valuation of health states using MNL models with interactions across multiple contexts and regions. However, it also has some limitations. The use of self-reported data and simulated ‘contexts’, while informative, might not fully capture the complexities and emotional depth of real-life palliative care needs situations. While the study covers multiple countries, the cultural diversity within each country is not specifically addressed, which might affect the generalizability of the findings to all sub-populations within those nations. Lastly, the study design assumes that the context provided in the vignettes was interpreted uniformly by all participants, which may not accurately reflect individual differences in perception and valuation of health states. These factors should be carefully considered when applying the study’s findings to policy and practice. 

In conclusion, this study contributes to the ongoing debate on the necessity of condition-specific value sets. While conventional practice involves using generic value sets for decision-making, our findings suggest potential variations in these values within a palliative care context. These findings highlight the need for a more nuanced valuation approach, possibly incorporating modifiers tailored to reflect the severity of palliative states, aligning with recent recommendations by NICE to prioritise health benefits in severe diseases, not limited to end-of-life conditions [8]. Alternatively, conducting sensitivity analyses with condition-specific value sets could offer valuable insights into decision-making frameworks within palliative care contexts, ensuring robust and equitable resource allocation decisions.

## Supplementary Information

Below is the link to the electronic supplementary material.

Supplementary file1 (DOCX 226 KB)
