# Supplementary material for: Does context matter? A discrete choice experiment investigating the impact of palliative context on EQ-5D-5L health state valuation
Source: Qual Life Res. 2025 Jul 29;34(10):2865–77. doi: 10.1007/s11136-025-04023-9 (PMC12535484; doi:10.1007/s11136-025-04023-9)
Supplement: Supplementary file 1 — Supplementary file1 (DOCX 226 KB) [file 11136_2025_4023_MOESM1_ESM.docx]

APPENDIX 1


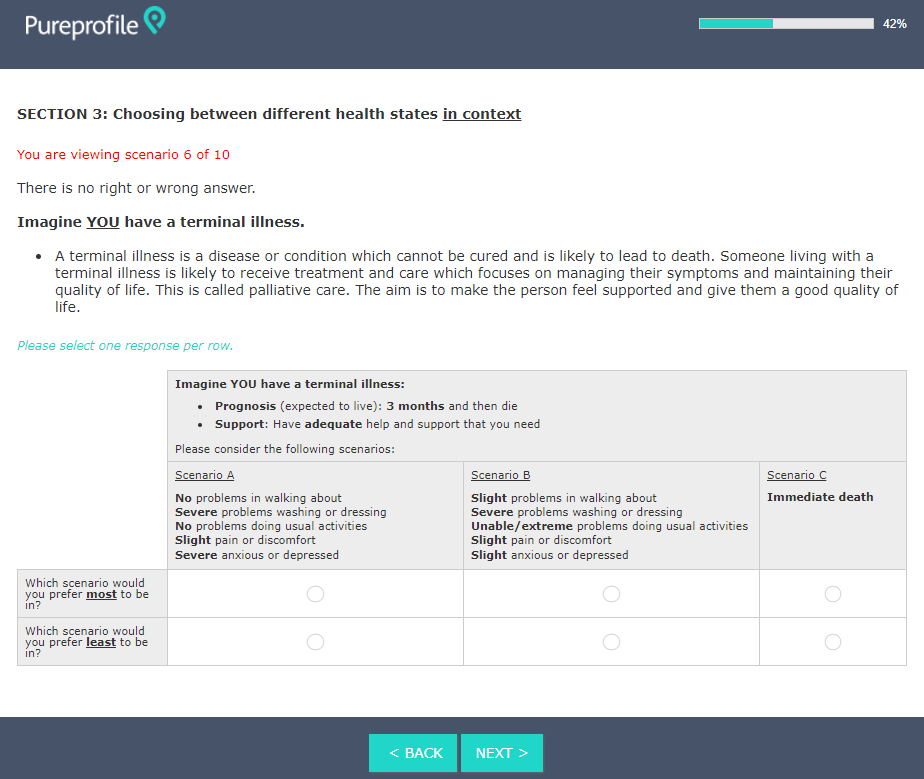


*Figure 1:* Example of a DCE with a palliative care context


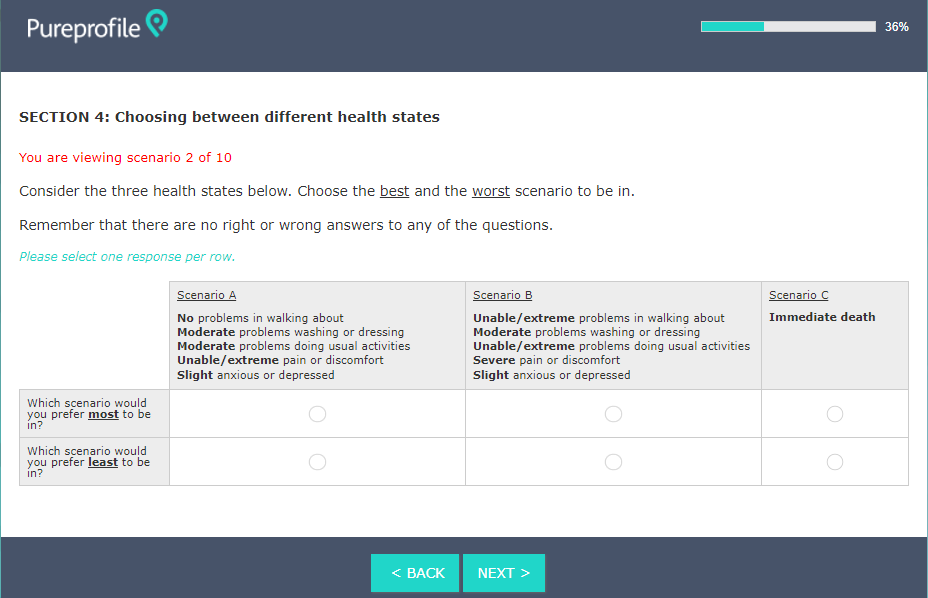


*Figure 2:* Example of a DCE without a context

APPENDIX 2

*Table 1.* Unadjusted and adjusted MNL of data before and after exclusion

|  | Unanchored estimates | | | | | | Anchored estimates | |
| --- | --- | --- | --- | --- | --- | --- | --- | --- |
|  | Before exclusion | | | After exclusion | | | Before  exclusion | After  exclusion |
|  | Coef. | SE | P. | Coef. | SE | P. | Coef. | Coef. |
| Mobility | | | | | | | | |
| MO2 | -0.225 | 0.013 | <0.001 | -0.235 | 0.014 | <0.001 | -0.085 | -0.077 |
| MO3 | -0.625 | 0.015 | <0.001 | -0.669 | 0.016 | <0.001 | -0.235 | -0.220 |
| MO4 | -0.539 | 0.021 | <0.001 | -0.648 | 0.022 | <0.001 | -0.203 | -0.213 |
| MO5 | -1.277 | 0.024 | <0.001 | -1.320 | 0.025 | <0.001 | -0.480 | -0.435 |
| Self-care | | | | | | | | |
| SC2 | -0.193 | 0.016 | <0.001 | -0.215 | 0.018 | <0.001 | -0.073 | -0.071 |
| SC3 | -0.159 | 0.018 | <0.001 | -0.170 | 0.019 | <0.001 | -0.060 | -0.056 |
| SC4 | -0.699 | 0.017 | <0.001 | -0.766 | 0.018 | <0.001 | -0.263 | -0.252 |
| SC5 | -0.825 | 0.018 | <0.001 | -0.926 | 0.019 | <0.001 | -0.310 | -0.305 |
| Usual activities | | | | | | | | |
| UA2 | -0.121 | 0.016 | <0.001 | -0.151 | 0.018 | <0.001 | -0.046 | -0.050 |
| UA3 | 0.067 | 0.017 | <0.001 | 0.027 | 0.018 | 0.145 | 0.025 | 0.009 |
| UA4 | -0.366 | 0.018 | <0.001 | -0.456 | 0.019 | <0.001 | -0.138 | -0.150 |
| UA5 | -0.707 | 0.017 | <0.001 | -0.810 | 0.019 | <0.001 | -0.266 | -0.267 |
| Pain/discomfort | | | | | | | | |
| PD2 | -0.132 | 0.016 | <0.001 | -0.158 | 0.018 | <0.001 | -0.050 | -0.052 |
| PD3 | 0.001 | 0.019 | 0.968 | -0.054 | 0.020 | 0.007 | 0.000 | -0.018 |
| PD4 | -0.636 | 0.019 | <0.001 | -0.768 | 0.021 | <0.001 | -0.239 | -0.253 |
| PD5 | -0.967 | 0.019 | <0.001 | -1.137 | 0.020 | <0.001 | -0.364 | -0.375 |
| Anxiety/depression | | | | | | | | |
| AD2 | -0.164 | 0.017 | <0.001 | -0.176 | 0.018 | <0.001 | -0.062 | -0.058 |
| AD3 | -0.027 | 0.020 | 0.191 | -0.075 | 0.022 | 0.001 | -0.010 | -0.025 |
| AD4 | -0.454 | 0.020 | <0.001 | -0.568 | 0.022 | <0.001 | -0.171 | -0.187 |
| AD5 | -0.786 | 0.021 | <0.001 | -0.938 | 0.022 | <0.001 | -0.296 | -0.309 |
| Death | -2.659 | 0.028 | <0.001 | -3.036 | 0.030 | <0.001 | -1.000 | -1.000 |
| AIC | 198953.3 | | | 171975.2 | | |  |  |
| Log-Likelihood | -99456 | | | -85967 | | |  |  |

Table 2a. Anchored MNL coefficients using Australian data

|  | Study arm 1 | | | | Study arm 2 | | | | Study arm 3 | | | | Study arm 4 | | | |
| --- | --- | --- | --- | --- | --- | --- | --- | --- | --- | --- | --- | --- | --- | --- | --- | --- |
|  | Context | | No context | | Context | | No context | | Context | | No context | | Context | | No context | |
|  | Coef. | SE | Coef. | SE | Coef. | SE | Coef. | SE | Coef. | SE | Coef. | SE | Coef. | SE | Coef. | SE |
| Mobility | | | | | | | | | | | | | | | | |
| MO2 | -0.095 | 0.028 | -0.070 | 0.023 | -0.077 | 0.023 | -0.069 | 0.024 | -0.092 | 0.023 | -0.077 | 0.023 | -0.077 | 0.022 | -0.093 | 0.023 |
| MO3 | -0.322 | 0.031 | -0.226 | 0.026 | -0.260 | 0.025 | -0.244 | 0.026 | -0.227 | 0.025 | -0.234 | 0.025 | -0.235 | 0.025 | -0.231 | 0.026 |
| MO4 | **-0.312** | 0.043 | -0.239 | 0.036 | -0.271 | 0.035 | **-0.223** | 0.036 | **-0.105** | 0.036 | **-0.181** | 0.036 | **-0.203** | 0.035 | **-0.184** | 0.036 |
| MO5 | -0.606 | 0.052 | -0.467 | 0.041 | -0.465 | 0.042 | -0.496 | 0.042 | -0.456 | 0.042 | -0.486 | 0.042 | -0.394 | 0.038 | -0.453 | 0.040 |
| Self-care | | | | | | | | | | | | | | | | |
| SC2 | -0.111 | 0.035 | -0.051 | 0.029 | -0.096 | 0.029 | -0.098 | 0.029 | -0.054 | 0.029 | -0.059 | 0.029 | -0.126 | 0.028 | -0.096 | 0.028 |
| SC3 | **-0.098** | 0.036 | **-0.033** | 0.030 | **-0.047** | 0.030 | **-0.050** | 0.031 | **-0.024** | 0.030 | **0.007** | 0.030 | **-0.077** | 0.030 | **-0.074** | 0.030 |
| SC4 | -0.348 | 0.035 | -0.285 | 0.029 | -0.252 | 0.029 | -0.255 | 0.030 | -0.268 | 0.030 | -0.269 | 0.030 | -0.301 | 0.028 | -0.277 | 0.029 |
| SC5 | -0.409 | 0.039 | -0.321 | 0.031 | -0.305 | 0.032 | -0.308 | 0.032 | -0.307 | 0.032 | -0.327 | 0.032 | -0.316 | 0.030 | -0.357 | 0.031 |
| Usual activities | | | | | | | | | | | | | | | | |
| UA2 | **0.007** | 0.035 | -0.044 | 0.029 | -0.056 | 0.029 | -0.047 | 0.029 | -0.034 | 0.029 | -0.067 | 0.029 | -0.033 | 0.027 | -0.059 | 0.028 |
| UA3 | **0.072** | 0.036 | **0.015** | 0.030 | **0.001** | 0.030 | **0.026** | 0.030 | **0.018** | 0.030 | **0.011** | 0.030 | **0.027** | 0.028 | **0.013** | 0.029 |
| UA4 | -0.104 | 0.038 | -0.113 | 0.031 | -0.168 | 0.032 | -0.158 | 0.032 | -0.180 | 0.032 | -0.159 | 0.032 | -0.115 | 0.030 | -0.136 | 0.031 |
| UA5 | -0.348 | 0.039 | -0.314 | 0.031 | -0.292 | 0.031 | -0.329 | 0.032 | -0.270 | 0.030 | -0.289 | 0.030 | -0.219 | 0.029 | -0.270 | 0.030 |
| Pain/discomfort | | | | | | | | | | | | | | | | |
| PD2 | -0.074 | 0.035 | -0.054 | 0.029 | -0.047 | 0.028 | -0.017 | 0.029 | -0.069 | 0.028 | -0.072 | 0.028 | -0.075 | 0.027 | **0.012** | 0.028 |
| PD3 | **-0.027** | 0.040 | **-0.035** | 0.033 | -0.074 | 0.032 | **0.002** | 0.033 | **-0.065** | 0.032 | **-0.019** | 0.032 | **-0.010** | 0.031 | **0.078** | 0.032 |
| PD4 | -0.315 | 0.041 | -0.295 | 0.034 | -0.276 | 0.033 | -0.256 | 0.034 | -0.293 | 0.033 | -0.268 | 0.033 | -0.279 | 0.032 | -0.242 | 0.033 |
| PD5 | -0.465 | 0.041 | -0.422 | 0.033 | -0.384 | 0.033 | -0.386 | 0.033 | -0.380 | 0.032 | -0.398 | 0.032 | -0.389 | 0.031 | -0.386 | 0.032 |
| Anxiety/depression | | | | | | | | | | | | | | | | |
| AD2 | -0.041 | 0.036 | -0.023 | 0.029 | -0.145 | 0.030 | -0.078 | 0.030 | -0.086 | 0.029 | -0.054 | 0.029 | -0.030 | 0.028 | -0.030 | 0.029 |
| AD3 | **0.017** | 0.043 | **-0.011** | 0.035 | **-0.105** | 0.035 | **-0.048** | 0.036 | **-0.058** | 0.035 | **-0.006** | 0.035 | **0.010** | 0.034 | **0.062** | 0.035 |
| AD4 | -0.167 | 0.043 | -0.143 | 0.035 | -0.226 | 0.035 | -0.176 | 0.036 | -0.179 | 0.035 | -0.144 | 0.035 | -0.125 | 0.034 | -0.129 | 0.034 |
| AD5 | -0.303 | 0.044 | -0.275 | 0.036 | -0.369 | 0.036 | -0.368 | 0.037 | -0.336 | 0.036 | -0.292 | 0.036 | -0.249 | 0.034 | -0.262 | 0.035 |
| AIC | 10638 |  | 10016 |  | 9792 |  | 9500 |  | 9690 |  | 9487 |  | 9634 |  | 9427 |  |
| BIC | 10777 |  | 10155 |  | 9929 |  | 9637 |  | 9828 |  | 9624 |  | 9772 |  | 9565 |  |
| LL | -5298 |  | -4987 |  | -4875 |  | -4729 |  | -4824 |  | -4722 |  | -4796 |  | -4692 |  |

Table 2b. Anchored MNL coefficients using Irish data

|  | Study arm 1 | | | | Study arm 2 | | | | Study arm 3 | | | | Study arm 4 | | | |
| --- | --- | --- | --- | --- | --- | --- | --- | --- | --- | --- | --- | --- | --- | --- | --- | --- |
|  | Context | | No context | | Context | | No context | | Context | | No context | | Context | | No context | |
|  | Coef. | SE | Coef. | SE | Coef. | SE | Coef. | SE | Coef. | SE | Coef. | SE | Coef. | SE | Coef. | SE |
| Mobility | | | | | | | | | | | | | | | | |
| MO2 | -0.095 | 0.036 | -0.076 | 0.030 | -0.095 | 0.031 | -0.080 | 0.030 | -0.076 | 0.033 | -0.147 | 0.028 | -0.044 | 0.030 | -0.058 | 0.033 |
| MO3 | -0.223 | 0.039 | -0.195 | 0.033 | -0.208 | 0.035 | -0.206 | 0.034 | -0.175 | 0.037 | -0.235 | 0.031 | -0.205 | 0.033 | -0.121 | 0.036 |
| MO4 | **-0.134** | 0.055 | **-0.055** | 0.047 | **-0.165** | 0.049 | **-0.155** | 0.048 | **-0.160** | 0.051 | **-0.167** | 0.043 | -0.233 | 0.048 | -0.181 | 0.052 |
| MO5 | -0.555 | 0.066 | -0.374 | 0.052 | -0.455 | 0.055 | -0.392 | 0.052 | -0.463 | 0.057 | -0.464 | 0.048 | -0.415 | 0.052 | -0.450 | 0.056 |
| Self-care | | | | | | | | | | | | | | | | |
| SC2 | -0.102 | 0.043 | -0.060 | 0.036 | -0.116 | 0.039 | -0.092 | 0.037 | -0.109 | 0.041 | -0.052 | 0.034 | -0.016 | 0.038 | **0.064** | 0.041 |
| SC3 | -0.123 | 0.046 | -0.064 | 0.039 | **-0.054** | 0.041 | **-0.055** | 0.040 | **-0.055** | 0.044 | -0.067 | 0.037 | **0.003** | 0.040 | **0.061** | 0.043 |
| SC4 | -0.287 | 0.045 | -0.215 | 0.038 | -0.240 | 0.039 | -0.262 | 0.038 | -0.258 | 0.042 | -0.207 | 0.035 | -0.261 | 0.039 | -0.121 | 0.042 |
| SC5 | **-0.284** | 0.047 | -0.229 | 0.039 | **-0.204** | 0.039 | **-0.222** | 0.038 | -0.378 | 0.046 | -0.256 | 0.037 | -0.267 | 0.040 | -0.158 | 0.043 |
| Usual activities | | | | | | | | | | | | | | | | |
| UA2 | -0.021 | 0.043 | -0.046 | 0.037 | -0.065 | 0.038 | -0.023 | 0.037 | -0.090 | 0.041 | -0.054 | 0.034 | -0.085 | 0.037 | -0.052 | 0.040 |
| UA3 | **0.098** | 0.045 | **0.007** | 0.038 | **0.025** | 0.040 | **0.037** | 0.039 | **-0.015** | 0.043 | **-0.001** | 0.036 | **-0.002** | 0.039 | **0.123** | 0.042 |
| UA4 | -0.106 | 0.047 | -0.135 | 0.040 | -0.121 | 0.042 | -0.094 | 0.041 | -0.187 | 0.045 | -0.113 | 0.037 | -0.174 | 0.041 | -0.145 | 0.044 |
| UA5 | -0.235 | 0.045 | -0.261 | 0.038 | -0.169 | 0.039 | -0.135 | 0.038 | -0.251 | 0.043 | -0.179 | 0.035 | -0.267 | 0.039 | -0.230 | 0.042 |
| Pain/discomfort | | | | | | | | | | | | | | | | |
| PD2 | -0.044 | 0.043 | -0.062 | 0.036 | -0.021 | 0.038 | -0.041 | 0.037 | -0.052 | 0.041 | -0.037 | 0.034 | -0.020 | 0.037 | -0.107 | 0.040 |
| PD3 | **0.040** | 0.049 | **0.011** | 0.042 | **0.087** | 0.043 | **0.067** | 0.042 | **0.000** | 0.046 | **0.043** | 0.039 | **0.013** | 0.043 | **-0.051** | 0.046 |
| PD4 | -0.197 | 0.050 | -0.193 | 0.043 | -0.105 | 0.045 | -0.135 | 0.043 | -0.249 | 0.048 | -0.182 | 0.040 | -0.195 | 0.044 | -0.274 | 0.047 |
| PD5 | -0.315 | 0.048 | -0.281 | 0.041 | -0.305 | 0.043 | -0.263 | 0.041 | -0.346 | 0.045 | -0.304 | 0.038 | -0.310 | 0.041 | -0.370 | 0.044 |
| Anxiety/depression | | | | | | | | | | | | | | | | |
| AD2 | -0.128 | 0.044 | -0.065 | 0.037 | -0.120 | 0.039 | -0.025 | 0.038 | -0.004 | 0.042 | -0.091 | 0.035 | -0.029 | 0.038 | -0.095 | 0.041 |
| AD3 | **-0.062** | 0.054 | -0.072 | 0.046 | **-0.035** | 0.047 | **0.055** | 0.046 | **0.043** | 0.051 | **-0.026** | 0.043 | -0.030 | 0.046 | **-0.030** | 0.050 |
| AD4 | -0.261 | 0.053 | -0.253 | 0.045 | -0.197 | 0.047 | -0.160 | 0.046 | -0.058 | 0.050 | -0.156 | 0.042 | -0.182 | 0.045 | -0.222 | 0.049 |
| AD5 | -0.305 | 0.054 | -0.326 | 0.046 | -0.260 | 0.048 | -0.215 | 0.046 | -0.236 | 0.051 | -0.268 | 0.043 | -0.363 | 0.047 | -0.371 | 0.050 |
| AIC | 6327 |  | 6065 |  | 5986 |  | 5790 |  | 6082 |  | 5858 |  | 6019 |  | 6062 |  |
| BIC | 6455 |  | 6192 |  | 6113 |  | 5917 |  | 6210 |  | 5985 |  | 6147 |  | 6190 |  |
| LL | -3143 |  | -3011 |  | -2972 |  | -2874 |  | -3020 |  | -2908 |  | -2989 |  | -3010 |  |

Table 2c. Anchored MNL coefficients using UK data

|  | Study arm 1 | | | | Study arm 2 | | | | Study arm 3 | | | | Study arm 4 | | | |
| --- | --- | --- | --- | --- | --- | --- | --- | --- | --- | --- | --- | --- | --- | --- | --- | --- |
|  | Context | | No context | | Context | | No context | | Context | | No context | | Context | | No context | |
|  | Coef. | SE | Coef. | SE | Coef. | SE | Coef. | SE | Coef. | SE | Coef. | SE | Coef. | SE | Coef. | SE |
| Mobility | | | | | | | | | | | | | | | | |
| MO2 | -0.077 | 0.026 | -0.085 | 0.021 | -0.134 | 0.022 | -0.089 | 0.019 | -0.097 | 0.023 | -0.083 | 0.021 | -0.074 | 0.021 | -0.053 | 0.020 |
| MO3 | -0.252 | 0.028 | -0.235 | 0.024 | -0.286 | 0.024 | -0.229 | 0.021 | -0.283 | 0.026 | -0.276 | 0.024 | -0.213 | 0.023 | -0.182 | 0.022 |
| MO4 | **-0.218** | 0.040 | -0.242 | 0.033 | -0.292 | 0.034 | **-0.180** | 0.030 | **-0.245** | 0.035 | **-0.261** | 0.033 | **-0.193** | 0.033 | **-0.111** | 0.031 |
| MO5 | -0.607 | 0.048 | -0.532 | 0.039 | -0.516 | 0.039 | -0.506 | 0.036 | -0.496 | 0.041 | -0.444 | 0.037 | -0.475 | 0.037 | -0.471 | 0.036 |
| Self-care | | | | | | | | | | | | | | | | |
| SC2 | -0.055 | 0.032 | -0.047 | 0.026 | -0.084 | 0.027 | -0.047 | 0.024 | -0.078 | 0.028 | -0.070 | 0.026 | -0.062 | 0.026 | -0.100 | 0.025 |
| SC3 | -0.110 | 0.033 | -0.058 | 0.028 | -0.109 | 0.028 | -0.055 | 0.025 | -0.094 | 0.030 | **-0.044** | 0.028 | -0.095 | 0.028 | **-0.090** | 0.026 |
| SC4 | -0.262 | 0.033 | -0.262 | 0.027 | -0.263 | 0.027 | -0.238 | 0.025 | -0.284 | 0.029 | -0.222 | 0.027 | -0.288 | 0.027 | -0.293 | 0.026 |
| SC5 | -0.371 | 0.036 | -0.307 | 0.029 | -0.333 | 0.029 | -0.289 | 0.026 | -0.356 | 0.032 | -0.324 | 0.029 | -0.354 | 0.029 | -0.290 | 0.027 |
| Usual activities | | | | | | | | | | | | | | | | |
| UA2 | -0.098 | 0.032 | -0.078 | 0.026 | -0.058 | 0.026 | -0.024 | 0.024 | -0.026 | 0.028 | -0.010 | 0.026 | **0.019** | 0.026 | -0.070 | 0.025 |
| UA3 | **-0.004** | 0.033 | **0.013** | 0.027 | **0.014** | 0.027 | **0.053** | 0.025 | **0.033** | 0.030 | **0.049** | 0.027 | **0.062** | 0.027 | **-0.008** | 0.026 |
| UA4 | -0.176 | 0.035 | -0.149 | 0.029 | -0.147 | 0.029 | -0.121 | 0.026 | -0.124 | 0.032 | -0.108 | 0.029 | -0.112 | 0.028 | -0.138 | 0.027 |
| UA5 | -0.352 | 0.034 | -0.266 | 0.027 | -0.296 | 0.028 | -0.267 | 0.025 | -0.270 | 0.030 | -0.247 | 0.027 | -0.222 | 0.027 | -0.264 | 0.025 |
| Pain/discomfort | | | | | | | | | | | | | | | | |
| PD2 | -0.016 | 0.032 | -0.024 | 0.026 | -0.055 | 0.026 | -0.064 | 0.023 | -0.045 | 0.028 | -0.104 | 0.026 | -0.052 | 0.026 | -0.023 | 0.025 |
| PD3 | **0.025** | 0.036 | **0.006** | 0.030 | **-0.001** | 0.030 | **-0.037** | 0.027 | **0.060** | 0.032 | **0.026** | 0.029 | **-0.039** | 0.029 | **0.007** | 0.028 |
| PD4 | -0.266 | 0.037 | -0.198 | 0.031 | -0.223 | 0.031 | -0.248 | 0.028 | -0.201 | 0.033 | -0.236 | 0.030 | -0.247 | 0.030 | -0.210 | 0.029 |
| PD5 | -0.403 | 0.036 | -0.310 | 0.029 | -0.361 | 0.030 | -0.366 | 0.027 | -0.328 | 0.031 | -0.311 | 0.029 | -0.408 | 0.029 | -0.369 | 0.028 |
| Anxiety/depression | | | | | | | | | | | | | | | | |
| AD2 | -0.047 | 0.032 | -0.065 | 0.026 | -0.053 | 0.027 | -0.093 | 0.024 | -0.055 | 0.029 | -0.053 | 0.026 | -0.075 | 0.026 | -0.022 | 0.025 |
| AD3 | **-0.004** | 0.039 | **-0.004** | 0.032 | **0.007** | 0.033 | **-0.013** | 0.029 | **-0.022** | 0.035 | **0.001** | 0.032 | **0.037** | 0.032 | **0.009** | 0.031 |
| AD4 | -0.185 | 0.039 | -0.179 | 0.032 | -0.191 | 0.032 | -0.203 | 0.029 | -0.179 | 0.034 | -0.163 | 0.032 | -0.137 | 0.032 | -0.147 | 0.030 |
| AD5 | -0.358 | 0.040 | -0.265 | 0.033 | -0.296 | 0.034 | -0.305 | 0.030 | -0.285 | 0.035 | -0.293 | 0.032 | -0.269 | 0.032 | -0.243 | 0.031 |
| AIC | 9203 |  | 8565 |  | 9665 |  | 8994 |  | 9437 |  | 9087 |  | 8764 |  | 8553 |  |
| BIC | 9339 |  | 8702 |  | 9803 |  | 9132 |  | 9574 |  | 9225 |  | 8900 |  | 8690 |  |
| LL | -4581 |  | -4262 |  | -4811 |  | -4476 |  | -4697 |  | -4523 |  | -4361 |  | -4255 |  |

**APPENDIX 3**

*Table 1a.* A MNL with main effects and interactions between a 3-level categorical variable (no context, 3-month life expectancy or 2-year life expectancy) and each attribute level in the Australian sample

|  | Main effects | | | Interaction terms | | | | | |
| --- | --- | --- | --- | --- | --- | --- | --- | --- | --- |
|  |  | | | 3-month | | | 2-year | | |
|  | Coef. | SE | P. | Coef. | SE | P. | Coef. | SE | P. |
| MO2 | -0.203 | 0.031 | **<0.001** | 0.005 | 0.052 | 0.925 | -0.021 | 0.053 | 0.691 |
| MO3 | -0.411 | 0.028 | **<0.001** | -0.071 | 0.048 | 0.138 | 0.017 | 0.048 | 0.714 |
| MO4 | 0.068 | 0.039 | 0.084 | -0.073 | 0.069 | 0.285 | 0.134 | 0.068 | 0.050 |
| MO5 | -0.697 | 0.058 | **<0.001** | 0.132 | 0.101 | 0.191 | -0.014 | 0.100 | 0.890 |
| SC2 | -0.201 | 0.038 | **<0.001** | -0.042 | 0.064 | 0.516 | -0.043 | 0.065 | 0.512 |
| SC3 | 0.102 | 0.036 | **0.004** | -0.025 | 0.061 | 0.675 | 0.002 | 0.061 | 0.978 |
| SC4 | -0.616 | 0.037 | **<0.001** | 0.083 | 0.063 | 0.182 | -0.007 | 0.063 | 0.918 |
| SC5 | -0.151 | 0.043 | **0.001** | 0.016 | 0.075 | 0.835 | 0.074 | 0.075 | 0.321 |
| UA2 | -0.140 | 0.038 | **<0.001** | 0.078 | 0.065 | 0.226 | 0.053 | 0.065 | 0.415 |
| UA3 | 0.181 | 0.033 | **<0.001** | -0.039 | 0.057 | 0.490 | -0.036 | 0.057 | 0.533 |
| UA4 | -0.411 | 0.039 | **<0.001** | 0.007 | 0.066 | 0.920 | -0.039 | 0.067 | 0.556 |
| UA5 | -0.418 | 0.043 | **<0.001** | -0.005 | 0.074 | 0.950 | 0.160 | 0.073 | **0.030** |
| PD2 | -0.087 | 0.037 | **0.019** | -0.057 | 0.064 | 0.371 | -0.104 | 0.064 | 0.105 |
| PD3 | 0.102 | 0.034 | **0.003** | -0.084 | 0.058 | 0.148 | -0.010 | 0.059 | 0.870 |
| PD4 | -0.715 | 0.040 | **<0.001** | 0.146 | 0.069 | **0.035** | 0.046 | 0.070 | 0.514 |
| PD5 | -0.343 | 0.041 | **<0.001** | 0.044 | 0.071 | 0.537 | 0.084 | 0.070 | 0.234 |
| AD2 | -0.120 | 0.038 | **0.002** | -0.107 | 0.066 | 0.104 | -0.037 | 0.066 | 0.581 |
| AD3 | 0.119 | 0.037 | **0.001** | -0.004 | 0.063 | 0.949 | -0.030 | 0.064 | 0.643 |
| AD4 | -0.387 | 0.040 | **<0.001** | 0.034 | 0.068 | 0.613 | 0.047 | 0.069 | 0.495 |
| AD5 | -0.394 | 0.038 | **<0.001** | 0.062 | 0.066 | 0.348 | 0.021 | 0.066 | 0.749 |
| Death | -2.625 | 0.064 | **<0.001** | 0.270 | 0.110 | **0.014** | -0.051 | 0.111 | 0.647 |
| LL |  |  |  |  |  |  |  |  | -38991 |
| AIC |  |  |  |  |  |  |  |  | 78108 |
| BIC |  |  |  |  |  |  |  |  | 78652 |

Note. Significant terms are bolded. Coef: coefficient estimate; SE: standard error; LL: log-likelihood; AIC: Akaike’s information criteria; BIC: Bayesian information criteria;

*Table 1b.* A MNL with main effects and interactions between a 3-level categorical variable (no context, 3-month life expectancy or 2-year life expectancy) and each attribute level in the Irish sample

|  | Main effects | | | Interaction terms | | | | | |
| --- | --- | --- | --- | --- | --- | --- | --- | --- | --- |
|  |  | | | 3-month | | | 2-year | | |
|  | Coef. | SE | P. | Coef. | SE | P. | Coef. | SE | P. |
| MO2 | -0.235 | 0.039 | **<0.001** | 0.016 | 0.067 | 0.812 | 0.090 | 0.067 | 0.181 |
| MO3 | -0.254 | 0.034 | **<0.001** | -0.027 | 0.060 | 0.656 | -0.062 | 0.060 | 0.295 |
| MO4 | 0.138 | 0.049 | **0.005** | 0.014 | 0.085 | 0.870 | -0.155 | 0.085 | 0.070 |
| MO5 | -0.724 | 0.073 | **<0.001** | -0.092 | 0.129 | 0.475 | 0.143 | 0.124 | 0.250 |
| SC2 | -0.103 | 0.047 | **0.031** | -0.150 | 0.082 | 0.067 | -0.051 | 0.082 | 0.533 |
| SC3 | 0.015 | 0.045 | 0.734 | 0.036 | 0.077 | 0.643 | 0.079 | 0.077 | 0.307 |
| SC4 | -0.438 | 0.047 | **<0.001** | 0.030 | 0.080 | 0.710 | -0.134 | 0.081 | 0.098 |
| SC5 | -0.034 | 0.054 | 0.535 | 0.085 | 0.094 | 0.365 | -0.112 | 0.095 | 0.237 |
| UA2 | -0.113 | 0.047 | **0.017** | 0.013 | 0.081 | 0.873 | -0.094 | 0.082 | 0.252 |
| UA3 | 0.211 | 0.042 | **<0.001** | 0.028 | 0.073 | 0.706 | -0.020 | 0.073 | 0.783 |
| UA4 | -0.410 | 0.049 | **<0.001** | 0.005 | 0.085 | 0.954 | -0.008 | 0.085 | 0.925 |
| UA5 | -0.203 | 0.052 | **<0.001** | 0.007 | 0.090 | 0.937 | 0.008 | 0.091 | 0.928 |
| PD2 | -0.158 | 0.047 | **0.001** | 0.084 | 0.081 | 0.302 | 0.073 | 0.082 | 0.371 |
| PD3 | 0.210 | 0.043 | **<0.001** | 0.017 | 0.074 | 0.819 | -0.107 | 0.075 | 0.152 |
| PD4 | -0.554 | 0.051 | **<0.001** | 0.052 | 0.087 | 0.546 | 0.001 | 0.088 | 0.991 |
| PD5 | -0.280 | 0.050 | **<0.001** | -0.094 | 0.088 | 0.284 | 0.022 | 0.087 | 0.800 |
| AD2 | -0.171 | 0.048 | **<0.001** | -0.113 | 0.083 | 0.172 | 0.131 | 0.083 | 0.116 |
| AD3 | 0.134 | 0.047 | **0.004** | 0.043 | 0.080 | 0.595 | -0.071 | 0.081 | 0.376 |
| AD4 | -0.460 | 0.050 | **<0.001** | 0.043 | 0.087 | 0.623 | 0.146 | 0.087 | 0.091 |
| AD5 | -0.250 | 0.048 | **<0.001** | 0.121 | 0.083 | 0.144 | -0.185 | 0.083 | **0.026** |
| Death | -2.561 | 0.081 | **<0.001** | 0.251 | 0.140 | 0.073 | 0.142 | 0.141 | 0.311 |
| LL |  |  |  |  |  |  |  |  | -24002 |
| AIC |  |  |  |  |  |  |  |  | 48130 |
| BIC |  |  |  |  |  |  |  |  | 48644 |

Note. Significant terms are bolded. Coef: coefficient estimate; SE: standard error; LL: log-likelihood; AIC: Akaike’s information criteria; BIC: Bayesian information criteria;

*Table 1c.* A MNL with main effects and interactions between a 3-level categorical (no context, 3-month life expectancy or 2-year life expectancy) and each attribute level in the UK sample

|  | Main effects | | | Interaction terms | | | | | |
| --- | --- | --- | --- | --- | --- | --- | --- | --- | --- |
|  |  | | | 3-month | | | 2-year | | |
|  | Coef. | SE | P. | Coef. | SE | P. | Coef. | SE | P. |
| MO2 | -0.244 | 0.032 | **<0.001** | -0.043 | 0.054 | 0.424 | 0.000 | 0.055 | 0.997 |
| MO3 | -0.470 | 0.028 | **<0.001** | 0.039 | 0.048 | 0.420 | 0.013 | 0.048 | 0.794 |
| MO4 | 0.101 | 0.040 | **0.012** | -0.071 | 0.070 | 0.309 | -0.021 | 0.069 | 0.764 |
| MO5 | -0.896 | 0.061 | **<0.001** | 0.108 | 0.105 | 0.306 | 0.138 | 0.103 | 0.180 |
| SC2 | -0.203 | 0.039 | **<0.001** | 0.014 | 0.066 | 0.833 | 0.002 | 0.066 | 0.971 |
| SC3 | 0.014 | 0.036 | 0.693 | -0.116 | 0.062 | 0.063 | -0.083 | 0.062 | 0.185 |
| SC4 | -0.595 | 0.038 | **<0.001** | 0.187 | 0.065 | **0.004** | 0.052 | 0.066 | 0.430 |
| SC5 | -0.153 | 0.045 | **0.001** | -0.081 | 0.078 | 0.300 | -0.044 | 0.079 | 0.574 |
| UA2 | -0.140 | 0.039 | **<0.001** | -0.061 | 0.067 | 0.358 | 0.130 | 0.067 | 0.051 |
| UA3 | 0.222 | 0.035 | **<0.001** | -0.007 | 0.059 | 0.904 | -0.078 | 0.059 | 0.187 |
| UA4 | -0.485 | 0.040 | **<0.001** | 0.046 | 0.068 | 0.501 | 0.016 | 0.069 | 0.816 |
| UA5 | -0.408 | 0.043 | **<0.001** | -0.023 | 0.075 | 0.758 | 0.049 | 0.075 | 0.513 |
| PD2 | -0.169 | 0.039 | **<0.001** | 0.069 | 0.066 | 0.297 | 0.031 | 0.066 | 0.643 |
| PD3 | 0.168 | 0.036 | **<0.001** | -0.041 | 0.061 | 0.499 | -0.002 | 0.061 | 0.968 |
| PD4 | -0.693 | 0.042 | **<0.001** | 0.023 | 0.072 | 0.745 | 0.028 | 0.072 | 0.692 |
| PD5 | -0.364 | 0.042 | **<0.001** | -0.001 | 0.072 | 0.987 | -0.046 | 0.072 | 0.524 |
| AD2 | -0.184 | 0.039 | **<0.001** | 0.053 | 0.068 | 0.436 | -0.004 | 0.068 | 0.957 |
| AD3 | 0.174 | 0.039 | **<0.001** | -0.035 | 0.066 | 0.596 | 0.037 | 0.066 | 0.581 |
| AD4 | -0.528 | 0.042 | **<0.001** | 0.020 | 0.071 | 0.778 | 0.055 | 0.072 | 0.444 |
| AD5 | -0.321 | 0.040 | **<0.001** | -0.036 | 0.068 | 0.596 | -0.016 | 0.068 | 0.819 |
| Death | -3.106 | 0.067 | **<0.001** | 0.451 | 0.114 | **<0.001** | 0.265 | 0.115 | **0.021** |
| LL |  |  |  |  |  |  |  |  | -36047 |
| AIC |  |  |  |  |  |  |  |  | 72220 |
| BIC |  |  |  |  |  |  |  |  | 72762 |

Note. Significant terms are bolded. Coef: coefficient estimate; SE: standard error; LL: log-likelihood; AIC: Akaike’s information criteria; BIC: Bayesian information criteria;

*Table 2a.* A MNL with main effects and interactions between a 3-level categorical variable (no context, limited support or adequate support) and each attribute level in the Australian sample

|  | Main effects | | | Interaction terms | | | | | |
| --- | --- | --- | --- | --- | --- | --- | --- | --- | --- |
|  |  | | | Limited | | | Adequate | | |
|  | Coef. | SE | P. | Coef. | SE | P. | Coef. | SE | P. |
| MO2 | -0.203 | 0.031 | **<0.001** | -0.017 | 0.052 | 0.744 | -0.001 | 0.053 | 0.979 |
| MO3 | -0.411 | 0.028 | **<0.001** | -0.005 | 0.047 | 0.909 | -0.048 | 0.048 | 0.319 |
| MO4 | 0.068 | 0.039 | 0.084 | 0.095 | 0.068 | 0.163 | -0.041 | 0.069 | 0.548 |
| MO5 | -0.697 | 0.058 | **<0.001** | -0.068 | 0.101 | 0.499 | 0.190 | 0.100 | 0.057 |
| SC2 | -0.201 | 0.038 | **<0.001** | 0.013 | 0.065 | 0.842 | -0.098 | 0.065 | 0.133 |
| SC3 | 0.102 | 0.036 | **0.004** | -0.049 | 0.061 | 0.419 | 0.027 | 0.061 | 0.665 |
| SC4 | -0.616 | 0.037 | **<0.001** | 0.032 | 0.062 | 0.603 | 0.044 | 0.064 | 0.493 |
| SC5 | -0.151 | 0.043 | **0.001** | 0.035 | 0.075 | 0.636 | 0.054 | 0.075 | 0.476 |
| UA2 | -0.140 | 0.038 | **<0.001** | 0.105 | 0.065 | 0.103 | 0.024 | 0.065 | 0.709 |
| UA3 | 0.181 | 0.033 | **<0.001** | -0.045 | 0.056 | 0.425 | -0.028 | 0.057 | 0.626 |
| UA4 | -0.411 | 0.039 | **<0.001** | -0.033 | 0.066 | 0.620 | -0.005 | 0.067 | 0.943 |
| UA5 | -0.418 | 0.043 | **<0.001** | 0.047 | 0.073 | 0.526 | 0.114 | 0.074 | 0.123 |
| PD2 | -0.087 | 0.037 | **0.019** | -0.082 | 0.064 | 0.196 | -0.080 | 0.064 | 0.217 |
| PD3 | 0.102 | 0.034 | **0.003** | -0.046 | 0.058 | 0.425 | -0.050 | 0.059 | 0.401 |
| PD4 | -0.715 | 0.040 | **<0.001** | 0.118 | 0.069 | 0.088 | 0.081 | 0.070 | 0.248 |
| PD5 | -0.343 | 0.041 | **<0.001** | 0.074 | 0.071 | 0.297 | 0.056 | 0.071 | 0.430 |
| AD2 | -0.120 | 0.038 | **0.002** | -0.033 | 0.066 | 0.618 | -0.110 | 0.067 | 0.097 |
| AD3 | 0.119 | 0.037 | **0.001** | -0.014 | 0.063 | 0.818 | -0.014 | 0.064 | 0.827 |
| AD4 | -0.387 | 0.040 | **<0.001** | 0.034 | 0.068 | 0.622 | 0.043 | 0.069 | 0.529 |
| AD5 | -0.394 | 0.038 | **<0.001** | 0.045 | 0.066 | 0.499 | 0.040 | 0.066 | 0.548 |
| Death | -2.625 | 0.064 | **<0.001** | 0.285 | 0.110 | **0.010** | -0.050 | 0.111 | 0.656 |
| LL |  |  |  |  |  |  |  |  | -39031 |
| AIC |  |  |  |  |  |  |  |  | 78188 |
| BIC |  |  |  |  |  |  |  |  | 78732 |

Note. Significant terms are bolded. Coef: coefficient estimate; SE: standard error; LL: log-likelihood; AIC: Akaike’s information criteria; BIC: Bayesian information criteria;

*Table 2b.* A MNL with main effects and interactions between a 3-level categorical variable (no context, limited support or adequate support) and each attribute level in the Irish sample

|  | Main effects | | | Interaction terms | | | | | |
| --- | --- | --- | --- | --- | --- | --- | --- | --- | --- |
|  |  | | | Limited | | | Adequate | | |
|  | Coef. | SE | P. | Coef. | SE | P. | Coef. | SE | P. |
| MO2 | -0.235 | 0.039 | **<0.001** | 0.044 | 0.067 | 0.513 | 0.060 | 0.067 | 0.367 |
| MO3 | -0.254 | 0.034 | **<0.001** | -0.002 | 0.060 | 0.979 | -0.090 | 0.060 | 0.131 |
| MO4 | 0.138 | 0.049 | **0.005** | -0.023 | 0.085 | 0.788 | -0.119 | 0.086 | 0.166 |
| MO5 | -0.724 | 0.073 | **<0.001** | -0.072 | 0.127 | 0.569 | 0.133 | 0.127 | 0.293 |
| SC2 | -0.103 | 0.047 | **0.031** | -0.134 | 0.081 | 0.098 | -0.057 | 0.083 | 0.487 |
| SC3 | 0.015 | 0.045 | 0.734 | 0.025 | 0.076 | 0.744 | 0.083 | 0.078 | 0.286 |
| SC4 | -0.438 | 0.047 | **<0.001** | 0.027 | 0.080 | 0.732 | -0.125 | 0.081 | 0.124 |
| SC5 | -0.034 | 0.054 | 0.535 | -0.103 | 0.095 | 0.279 | 0.067 | 0.093 | 0.472 |
| UA2 | -0.113 | 0.047 | **0.017** | -0.015 | 0.082 | 0.855 | -0.068 | 0.081 | 0.405 |
| UA3 | 0.211 | 0.042 | **<0.001** | 0.002 | 0.073 | 0.978 | 0.007 | 0.073 | 0.925 |
| UA4 | -0.410 | 0.049 | **<0.001** | -0.004 | 0.084 | 0.958 | 0.008 | 0.086 | 0.929 |
| UA5 | -0.203 | 0.052 | **<0.001** | -0.014 | 0.090 | 0.872 | 0.028 | 0.091 | 0.759 |
| PD2 | -0.158 | 0.047 | **0.001** | 0.052 | 0.081 | 0.521 | 0.103 | 0.082 | 0.207 |
| PD3 | 0.210 | 0.043 | **<0.001** | -0.058 | 0.074 | 0.434 | -0.030 | 0.074 | 0.685 |
| PD4 | -0.554 | 0.051 | **<0.001** | 0.002 | 0.087 | 0.981 | 0.056 | 0.088 | 0.522 |
| PD5 | -0.280 | 0.050 | **<0.001** | 0.046 | 0.087 | 0.598 | -0.110 | 0.087 | 0.209 |
| AD2 | -0.171 | 0.048 | **<0.001** | 0.028 | 0.083 | 0.739 | -0.012 | 0.083 | 0.888 |
| AD3 | 0.134 | 0.047 | **0.004** | -0.005 | 0.080 | 0.947 | -0.027 | 0.081 | 0.737 |
| AD4 | -0.460 | 0.050 | **<0.001** | 0.129 | 0.087 | 0.137 | 0.067 | 0.087 | 0.444 |
| AD5 | -0.250 | 0.048 | **<0.001** | -0.007 | 0.083 | 0.930 | -0.055 | 0.083 | 0.507 |
| Death | -2.561 | 0.081 | **<0.001** | 0.327 | 0.139 | **0.019** | 0.066 | 0.141 | 0.640 |
| LL |  |  |  |  |  |  |  |  | -24003 |
| AIC |  |  |  |  |  |  |  |  | 48133 |
| BIC |  |  |  |  |  |  |  |  | 48646 |

Note. Significant terms are bolded. Coef: coefficient estimate; SE: standard error; LL: log-likelihood; AIC: Akaike’s information criteria; BIC: Bayesian information criteria;

*Table 2c.* A MNL with main effects and interactions between a 3-level categorical variable (no context, limited support or adequate support) and each attribute level in the UK sample

|  | Main effects | | | Interaction terms | | | | | |
| --- | --- | --- | --- | --- | --- | --- | --- | --- | --- |
|  |  | | | Limited | | | Adequate | | |
|  | Coef. | SE | P. | Coef. | SE | P. | Coef. | SE | P. |
| MO2 | -0.244 | 0.032 | **<0.001** | 0.019 | 0.054 | 0.728 | -0.062 | 0.054 | 0.256 |
| MO3 | -0.470 | 0.028 | **<0.001** | 0.005 | 0.049 | 0.918 | 0.043 | 0.048 | 0.366 |
| MO4 | 0.101 | 0.040 | **0.012** | -0.006 | 0.070 | 0.926 | -0.082 | 0.070 | 0.242 |
| MO5 | -0.896 | 0.061 | **<0.001** | 0.090 | 0.104 | 0.389 | 0.156 | 0.104 | 0.132 |
| SC2 | -0.203 | 0.039 | **<0.001** | 0.030 | 0.066 | 0.646 | -0.016 | 0.067 | 0.810 |
| SC3 | 0.014 | 0.036 | 0.693 | -0.100 | 0.062 | 0.107 | -0.098 | 0.062 | 0.117 |
| SC4 | -0.595 | 0.038 | **<0.001** | 0.150 | 0.065 | **0.021** | 0.090 | 0.065 | 0.166 |
| SC5 | -0.153 | 0.045 | **0.001** | -0.076 | 0.079 | 0.335 | -0.050 | 0.078 | 0.524 |
| UA2 | -0.140 | 0.039 | **<0.001** | -0.010 | 0.067 | 0.877 | 0.086 | 0.067 | 0.199 |
| UA3 | 0.222 | 0.035 | **<0.001** | -0.034 | 0.059 | 0.564 | -0.055 | 0.059 | 0.351 |
| UA4 | -0.485 | 0.040 | **<0.001** | 0.063 | 0.068 | 0.357 | -0.004 | 0.069 | 0.955 |
| UA5 | -0.408 | 0.043 | **<0.001** | 0.000 | 0.075 | 0.997 | 0.028 | 0.075 | 0.710 |
| PD2 | -0.169 | 0.039 | **<0.001** | 0.089 | 0.066 | 0.181 | 0.011 | 0.066 | 0.871 |
| PD3 | 0.168 | 0.036 | **<0.001** | 0.023 | 0.061 | 0.710 | -0.069 | 0.061 | 0.255 |
| PD4 | -0.693 | 0.042 | **<0.001** | -0.011 | 0.072 | 0.874 | 0.064 | 0.072 | 0.374 |
| PD5 | -0.364 | 0.042 | **<0.001** | 0.026 | 0.072 | 0.720 | -0.074 | 0.072 | 0.304 |
| AD2 | -0.184 | 0.039 | **<0.001** | 0.049 | 0.068 | 0.468 | -0.003 | 0.067 | 0.962 |
| AD3 | 0.174 | 0.039 | **<0.001** | -0.076 | 0.066 | 0.252 | 0.077 | 0.066 | 0.242 |
| AD4 | -0.528 | 0.042 | **<0.001** | 0.097 | 0.071 | 0.176 | -0.018 | 0.071 | 0.801 |
| AD5 | -0.321 | 0.040 | **<0.001** | -0.030 | 0.068 | 0.655 | -0.021 | 0.068 | 0.761 |
| Death | -3.106 | 0.067 | **<0.001** | 0.538 | 0.114 | **<0.001** | 0.184 | 0.115 | 0.109 |
| LL |  |  |  |  |  |  |  |  | -36063 |
| AIC |  |  |  |  |  |  |  |  | 72253 |
| BIC |  |  |  |  |  |  |  |  | 72795 |

Note. Significant terms are bolded. Coef: coefficient estimate; SE: standard error; LL: log-likelihood; AIC: Akaike’s information criteria; BIC: Bayesian information criteria;

*Table 3a.* A MNL with main effects and interactions between a 5-level categorical variable indicating the no context vignette and the four context vignettes and each attribute level in the Australian sample

|  | Main effects | | | Interaction terms | | | | | | | | | | | |
| --- | --- | --- | --- | --- | --- | --- | --- | --- | --- | --- | --- | --- | --- | --- | --- |
|  |  | | | 3mth_limited | | | 3mth_adequate | | | 2yr_limited | | | 2yr_adequate | | |
|  | Coef. | SE | P. | Coef. | SE | P. | Coef. | SE | P. | Coef. | SE | P. | Coef. | SE | P. |
| MO2 | -0.203 | 0.031 | **<0.001** | 0.005 | 0.066 | 0.935 | 0.000 | 0.068 | 0.997 | -0.038 | 0.068 | 0.573 | -0.007 | 0.068 | 0.915 |
| MO3 | -0.411 | 0.028 | **<0.001** | -0.065 | 0.061 | 0.291 | -0.076 | 0.062 | 0.224 | 0.055 | 0.061 | 0.366 | -0.018 | 0.062 | 0.775 |
| MO4 | 0.068 | 0.039 | 0.084 | -0.046 | 0.088 | 0.597 | -0.097 | 0.089 | 0.279 | 0.252 | 0.089 | **0.004** | 0.019 | 0.088 | 0.833 |
| MO5 | -0.697 | 0.058 | **<0.001** | 0.082 | 0.130 | 0.530 | 0.183 | 0.132 | 0.167 | -0.228 | 0.133 | 0.087 | 0.178 | 0.127 | 0.163 |
| SC2 | -0.201 | 0.038 | **<0.001** | -0.032 | 0.082 | 0.699 | -0.053 | 0.084 | 0.529 | 0.060 | 0.085 | 0.483 | -0.141 | 0.084 | 0.094 |
| SC3 | 0.102 | 0.036 | **0.004** | -0.074 | 0.077 | 0.337 | 0.026 | 0.079 | 0.746 | -0.026 | 0.079 | 0.747 | 0.030 | 0.079 | 0.705 |
| SC4 | -0.616 | 0.037 | **<0.001** | 0.092 | 0.079 | 0.244 | 0.074 | 0.082 | 0.367 | -0.027 | 0.081 | 0.743 | 0.005 | 0.083 | 0.952 |
| SC5 | -0.151 | 0.043 | **0.001** | 0.023 | 0.096 | 0.809 | 0.009 | 0.098 | 0.929 | 0.049 | 0.097 | 0.616 | 0.111 | 0.097 | 0.254 |
| UA2 | -0.140 | 0.038 | **<0.001** | 0.154 | 0.082 | 0.060 | -0.009 | 0.085 | 0.920 | 0.050 | 0.085 | 0.560 | 0.050 | 0.083 | 0.547 |
| UA3 | 0.181 | 0.033 | **<0.001** | -0.045 | 0.072 | 0.529 | -0.031 | 0.074 | 0.674 | -0.043 | 0.073 | 0.560 | -0.019 | 0.074 | 0.801 |
| UA4 | -0.411 | 0.039 | **<0.001** | 0.044 | 0.083 | 0.595 | -0.036 | 0.086 | 0.679 | -0.112 | 0.087 | 0.197 | 0.026 | 0.086 | 0.765 |
| UA5 | -0.418 | 0.043 | **<0.001** | -0.092 | 0.094 | 0.328 | 0.090 | 0.097 | 0.354 | 0.181 | 0.096 | 0.059 | 0.135 | 0.094 | 0.149 |
| PD2 | -0.087 | 0.037 | **0.019** | -0.067 | 0.082 | 0.414 | -0.036 | 0.083 | 0.663 | -0.095 | 0.083 | 0.250 | -0.118 | 0.083 | 0.157 |
| PD3 | 0.102 | 0.034 | **0.003** | -0.004 | 0.073 | 0.957 | -0.174 | 0.076 | **0.022** | -0.090 | 0.076 | 0.237 | 0.075 | 0.077 | 0.327 |
| PD4 | -0.715 | 0.040 | **<0.001** | 0.112 | 0.088 | 0.205 | 0.180 | 0.090 | **0.046** | 0.113 | 0.090 | 0.207 | -0.017 | 0.091 | 0.852 |
| PD5 | -0.343 | 0.041 | **<0.001** | 0.030 | 0.091 | 0.744 | 0.056 | 0.093 | 0.546 | 0.115 | 0.092 | 0.210 | 0.044 | 0.090 | 0.623 |
| AD2 | -0.120 | 0.038 | **0.002** | 0.034 | 0.084 | 0.683 | -0.265 | 0.087 | **0.002** | -0.107 | 0.086 | 0.211 | 0.039 | 0.085 | 0.644 |
| AD3 | 0.119 | 0.037 | **0.001** | 0.003 | 0.080 | 0.971 | -0.013 | 0.082 | 0.874 | -0.044 | 0.082 | 0.592 | -0.011 | 0.083 | 0.895 |
| AD4 | -0.387 | 0.040 | **<0.001** | 0.001 | 0.087 | 0.988 | 0.066 | 0.089 | 0.456 | 0.068 | 0.089 | 0.444 | 0.019 | 0.089 | 0.826 |
| AD5 | -0.394 | 0.038 | **<0.001** | 0.111 | 0.084 | 0.191 | 0.015 | 0.086 | 0.861 | -0.021 | 0.086 | 0.810 | 0.056 | 0.085 | 0.508 |
| Death | -2.625 | 0.064 | **<0.001** | 0.535 | 0.139 | **<0.001** | -0.025 | 0.145 | 0.862 | -0.008 | 0.144 | 0.956 | -0.096 | 0.143 | 0.502 |
| LL |  |  |  |  |  |  |  |  |  |  |  |  |  |  | -38960 |
| AIC |  |  |  |  |  |  |  |  |  |  |  |  |  |  | 78177 |
| BIC |  |  |  |  |  |  |  |  |  |  |  |  |  |  | 78540 |

Note. Significant terms are bolded. Coef: coefficient estimate; SE: standard error; LL: log-likelihood; AIC: Akaike’s information criteria; BIC: Bayesian information criteria; 3mth_limited, a 3-month life expectancy with limited support vignette; 3mth_adequate, a 3-month life expectancy with adequate support vignette; 2yr_limited, a 2-year life expectancy with limited support vignette; 2yr_adequate, a 2-year life expectancy with adequate support vignette.

*Table 3b.* A MNL with main effects and interactions between a 5-level categorical variable indicating the no context vignette and the four context vignettes and each attribute level in the Irish sample

|  | Main effects | | | Interaction terms | | | | | | | | | | | |
| --- | --- | --- | --- | --- | --- | --- | --- | --- | --- | --- | --- | --- | --- | --- | --- |
|  |  | | | 3mth_limited | | | 3mth_adequate | | | 2yr_limited | | | 2yr_adequate | | |
|  | Coef. | SE | P. | Coef. | SE | P. | Coef. | SE | P. | Coef. | SE | P. | Coef. | SE | P. |
| MO2 | -0.235 | 0.039 | **<0.001** | 0.028 | 0.087 | 0.742 | -0.001 | 0.087 | 0.994 | 0.059 | 0.086 | 0.498 | 0.124 | 0.087 | 0.154 |
| MO3 | -0.254 | 0.034 | **<0.001** | -0.023 | 0.076 | 0.767 | -0.028 | 0.078 | 0.725 | 0.027 | 0.078 | 0.727 | -0.155 | 0.077 | **0.043** |
| MO4 | 0.138 | 0.049 | **0.005** | 0.055 | 0.110 | 0.616 | -0.030 | 0.111 | 0.789 | -0.104 | 0.109 | 0.340 | -0.211 | 0.113 | 0.062 |
| MO5 | -0.724 | 0.073 | **<0.001** | -0.187 | 0.170 | 0.272 | 0.004 | 0.166 | 0.982 | 0.022 | 0.159 | 0.888 | 0.262 | 0.162 | 0.106 |
| SC2 | -0.103 | 0.047 | **0.031** | -0.118 | 0.104 | 0.258 | -0.185 | 0.107 | 0.084 | -0.148 | 0.105 | 0.159 | 0.061 | 0.107 | 0.570 |
| SC3 | 0.015 | 0.045 | 0.734 | -0.061 | 0.098 | 0.535 | 0.138 | 0.102 | 0.175 | 0.110 | 0.099 | 0.267 | 0.035 | 0.100 | 0.729 |
| SC4 | -0.438 | 0.047 | **<0.001** | 0.083 | 0.103 | 0.420 | -0.024 | 0.105 | 0.822 | -0.032 | 0.105 | 0.756 | -0.235 | 0.104 | **0.024** |
| SC5 | -0.034 | 0.054 | 0.535 | 0.039 | 0.122 | 0.749 | 0.123 | 0.120 | 0.306 | -0.244 | 0.124 | 0.050 | 0.020 | 0.121 | 0.872 |
| UA2 | -0.113 | 0.047 | **0.017** | 0.068 | 0.105 | 0.519 | -0.049 | 0.105 | 0.639 | -0.096 | 0.107 | 0.369 | -0.103 | 0.105 | 0.326 |
| UA3 | 0.211 | 0.042 | **<0.001** | 0.047 | 0.095 | 0.622 | 0.014 | 0.095 | 0.882 | -0.037 | 0.095 | 0.700 | 0.001 | 0.093 | 0.993 |
| UA4 | -0.410 | 0.049 | **<0.001** | -0.032 | 0.108 | 0.770 | 0.047 | 0.112 | 0.676 | 0.012 | 0.109 | 0.915 | -0.027 | 0.110 | 0.802 |
| UA5 | -0.203 | 0.052 | **<0.001** | -0.076 | 0.116 | 0.514 | 0.085 | 0.117 | 0.466 | 0.055 | 0.116 | 0.635 | -0.035 | 0.120 | 0.770 |
| PD2 | -0.158 | 0.047 | **0.001** | 0.062 | 0.104 | 0.552 | 0.106 | 0.106 | 0.318 | 0.038 | 0.106 | 0.722 | 0.106 | 0.106 | 0.318 |
| PD3 | 0.210 | 0.043 | **<0.001** | -0.027 | 0.096 | 0.776 | 0.058 | 0.096 | 0.546 | -0.091 | 0.097 | 0.347 | -0.124 | 0.097 | 0.199 |
| PD4 | -0.554 | 0.051 | **<0.001** | 0.041 | 0.112 | 0.717 | 0.076 | 0.112 | 0.496 | -0.021 | 0.113 | 0.849 | 0.024 | 0.114 | 0.831 |
| PD5 | -0.280 | 0.050 | **<0.001** | 0.024 | 0.113 | 0.834 | -0.215 | 0.114 | 0.060 | 0.055 | 0.113 | 0.624 | -0.011 | 0.112 | 0.919 |
| AD2 | -0.171 | 0.048 | **<0.001** | -0.107 | 0.107 | 0.314 | -0.126 | 0.108 | 0.244 | 0.163 | 0.108 | 0.133 | 0.098 | 0.108 | 0.364 |
| AD3 | 0.134 | 0.047 | **0.004** | 0.010 | 0.103 | 0.921 | 0.075 | 0.104 | 0.469 | -0.026 | 0.104 | 0.801 | -0.136 | 0.105 | 0.194 |
| AD4 | -0.460 | 0.050 | **<0.001** | 0.030 | 0.112 | 0.791 | 0.060 | 0.115 | 0.599 | 0.228 | 0.112 | **0.043** | 0.073 | 0.111 | 0.514 |
| AD5 | -0.250 | 0.048 | **<0.001** | 0.154 | 0.107 | 0.149 | 0.093 | 0.107 | 0.386 | -0.162 | 0.108 | 0.133 | -0.211 | 0.108 | **0.049** |
| Death | -2.561 | 0.081 | **<0.001** | 0.395 | 0.178 | **0.026** | 0.079 | 0.184 | 0.667 | 0.249 | 0.181 | 0.169 | 0.018 | 0.182 | 0.920 |
| LL |  |  |  |  |  |  |  |  |  |  |  |  |  |  | -24023 |
| AIC |  |  |  |  |  |  |  |  |  |  |  |  |  |  | 48130 |
| BIC |  |  |  |  |  |  |  |  |  |  |  |  |  |  | 48472 |

Note. Significant terms are bolded. Coef: coefficient estimate; SE: standard error; LL: log-likelihood; AIC: Akaike’s information criteria; BIC: Bayesian information criteria; 3mth_limited, a 3-month life expectancy with limited support vignette; 3mth_adequate, a 3-month life expectancy with adequate support vignette; 2yr_limited, a 2-year life expectancy with limited support vignette; 2yr_adequate, a 2-year life expectancy with adequate support vignette.

*Table 3c.* A MNL with main effects and interactions between a 5-level categorical variable indicating the no context vignette and the four context vignettes and each attribute level in the UK sample

|  | Main effects | | | Interaction terms | | | | | | | | | | | |
| --- | --- | --- | --- | --- | --- | --- | --- | --- | --- | --- | --- | --- | --- | --- | --- |
|  |  | | | 3mth_limited | | | 3mth_adequate | | | 2yr_limited | | | 2yr_adequate | | |
|  | Coef. | SE | P. | Coef. | SE | P. | Coef. | SE | P. | Coef. | SE | P. | Coef. | SE | P. |
| MO2 | -0.244 | 0.032 | **<0.001** | 0.054 | 0.070 | 0.441 | -0.140 | 0.069 | **0.044** | -0.016 | 0.070 | 0.818 | 0.022 | 0.071 | 0.759 |
| MO3 | -0.470 | 0.028 | **<0.001** | 0.043 | 0.064 | 0.501 | 0.037 | 0.062 | 0.548 | -0.032 | 0.062 | 0.604 | 0.051 | 0.062 | 0.418 |
| MO4 | 0.101 | 0.040 | **0.012** | -0.019 | 0.092 | 0.841 | -0.119 | 0.090 | 0.185 | 0.001 | 0.088 | 0.993 | -0.043 | 0.091 | 0.639 |
| MO5 | -0.896 | 0.061 | **<0.001** | -0.056 | 0.139 | 0.687 | 0.258 | 0.134 | 0.054 | 0.222 | 0.131 | 0.090 | 0.047 | 0.134 | 0.723 |
| SC2 | -0.203 | 0.039 | **<0.001** | 0.069 | 0.086 | 0.423 | -0.036 | 0.085 | 0.671 | -0.007 | 0.084 | 0.930 | 0.015 | 0.087 | 0.865 |
| SC3 | 0.014 | 0.036 | 0.693 | -0.149 | 0.081 | 0.065 | -0.088 | 0.080 | 0.271 | -0.056 | 0.080 | 0.482 | -0.113 | 0.081 | 0.166 |
| SC4 | -0.595 | 0.038 | **<0.001** | 0.223 | 0.084 | **0.008** | 0.157 | 0.083 | 0.058 | 0.082 | 0.084 | 0.332 | 0.014 | 0.086 | 0.868 |
| SC5 | -0.153 | 0.045 | **0.001** | -0.115 | 0.102 | 0.259 | -0.047 | 0.099 | 0.634 | -0.041 | 0.101 | 0.685 | -0.048 | 0.103 | 0.642 |
| UA2 | -0.140 | 0.039 | **<0.001** | -0.099 | 0.087 | 0.257 | -0.026 | 0.085 | 0.762 | 0.071 | 0.085 | 0.403 | 0.198 | 0.087 | **0.023** |
| UA3 | 0.222 | 0.035 | **<0.001** | 0.007 | 0.077 | 0.926 | -0.017 | 0.075 | 0.825 | -0.065 | 0.075 | 0.384 | -0.093 | 0.077 | 0.231 |
| UA4 | -0.485 | 0.040 | **<0.001** | 0.063 | 0.088 | 0.473 | 0.026 | 0.087 | 0.762 | 0.062 | 0.089 | 0.482 | -0.040 | 0.090 | 0.656 |
| UA5 | -0.408 | 0.043 | **<0.001** | -0.023 | 0.097 | 0.811 | -0.020 | 0.096 | 0.836 | 0.017 | 0.096 | 0.862 | 0.078 | 0.097 | 0.422 |
| PD2 | -0.169 | 0.039 | **<0.001** | 0.130 | 0.087 | 0.132 | 0.013 | 0.084 | 0.879 | 0.047 | 0.084 | 0.579 | 0.012 | 0.087 | 0.888 |
| PD3 | 0.168 | 0.036 | **<0.001** | -0.068 | 0.079 | 0.386 | -0.015 | 0.077 | 0.844 | 0.114 | 0.078 | 0.144 | -0.129 | 0.080 | 0.106 |
| PD4 | -0.693 | 0.042 | **<0.001** | -0.018 | 0.094 | 0.848 | 0.059 | 0.091 | 0.519 | -0.009 | 0.091 | 0.924 | 0.067 | 0.093 | 0.474 |
| PD5 | -0.364 | 0.042 | **<0.001** | 0.028 | 0.094 | 0.767 | -0.029 | 0.092 | 0.754 | 0.024 | 0.092 | 0.790 | -0.123 | 0.094 | 0.190 |
| AD2 | -0.184 | 0.039 | **<0.001** | 0.069 | 0.088 | 0.432 | 0.034 | 0.087 | 0.699 | 0.036 | 0.087 | 0.678 | -0.041 | 0.088 | 0.643 |
| AD3 | 0.174 | 0.039 | **<0.001** | -0.069 | 0.085 | 0.416 | -0.003 | 0.084 | 0.967 | -0.084 | 0.085 | 0.323 | 0.161 | 0.086 | 0.062 |
| AD4 | -0.528 | 0.042 | **<0.001** | 0.086 | 0.093 | 0.354 | -0.038 | 0.090 | 0.673 | 0.105 | 0.092 | 0.251 | 0.006 | 0.093 | 0.949 |
| AD5 | -0.321 | 0.040 | **<0.001** | -0.101 | 0.090 | 0.260 | 0.023 | 0.087 | 0.791 | 0.036 | 0.087 | 0.676 | -0.078 | 0.089 | 0.384 |
| Death | -3.106 | 0.067 | **<0.001** | 0.658 | 0.148 | **<0.001** | 0.250 | 0.145 | 0.086 | 0.416 | 0.147 | **0.005** | 0.092 | 0.151 | 0.541 |
| LL |  |  |  |  |  |  |  |  |  |  |  |  |  |  | -36082 |
| AIC |  |  |  |  |  |  |  |  |  |  |  |  |  |  | 72249 |
| BIC |  |  |  |  |  |  |  |  |  |  |  |  |  |  | 72610 |

Note. Significant terms are bolded. Coef: coefficient estimate; SE: standard error; LL: log-likelihood; AIC: Akaike’s information criteria; BIC: Bayesian information criteria; 3mth_limited, a 3-month life expectancy with limited support vignette; 3mth_adequate, a 3-month life expectancy with adequate support vignette; 2yr_limited, a 2-year life expectancy with limited support vignette; 2yr_adequate, a 2-year life expectancy with adequate support vignette.

*Table 4a.* Separate MNLs with main effects and interactions between a dummy indicating a vignette with or without a context and each attribute level for each study arm in the Australian sample

|  | Study Arm 1 | | | Study Arm 2 | | | Study Arm 3 | | | | Study Arm 4 | | |
| --- | --- | --- | --- | --- | --- | --- | --- | --- | --- | --- | --- | --- | --- |
|  | Coef. | SE | P. | Coef. | SE | P. | Coef. | SE | P. | Coef. | | SE | P. |
| MO2 | -0.181 | 0.060 | **0.002** | -0.181 | 0.062 | **0.003** | -0.204 | 0.062 | **0.001** | -0.250 | | 0.062 | **<0.001** |
| MO3 | -0.402 | 0.055 | **<0.001** | -0.454 | 0.056 | **<0.001** | -0.416 | 0.055 | **<0.001** | -0.369 | | 0.055 | **<0.001** |
| MO4 | -0.031 | 0.078 | 0.691 | 0.053 | 0.080 | 0.507 | 0.140 | 0.080 | 0.079 | 0.128 | | 0.079 | 0.108 |
| MO5 | -0.588 | 0.114 | **<0.001** | -0.712 | 0.118 | **<0.001** | -0.809 | 0.120 | **<0.001** | -0.721 | | 0.114 | **<0.001** |
| SC2 | -0.132 | 0.074 | 0.073 | -0.255 | 0.076 | **0.001** | -0.156 | 0.077 | **0.041** | -0.257 | | 0.076 | **0.001** |
| SC3 | 0.047 | 0.070 | 0.509 | 0.124 | 0.071 | 0.082 | 0.175 | 0.072 | **0.015** | 0.060 | | 0.071 | 0.399 |
| SC4 | -0.649 | 0.072 | **<0.001** | -0.533 | 0.074 | **<0.001** | -0.732 | 0.073 | **<0.001** | -0.544 | | 0.075 | **<0.001** |
| SC5 | -0.091 | 0.085 | 0.287 | -0.137 | 0.088 | 0.119 | -0.153 | 0.088 | 0.080 | -0.215 | | 0.088 | **0.014** |
| UA2 | -0.113 | 0.073 | 0.126 | -0.123 | 0.076 | 0.108 | -0.178 | 0.077 | **0.020** | -0.157 | | 0.075 | **0.035** |
| UA3 | 0.151 | 0.065 | **0.021** | 0.192 | 0.067 | **0.004** | 0.206 | 0.066 | **0.002** | 0.191 | | 0.067 | **0.004** |
| UA4 | -0.330 | 0.075 | **<0.001** | -0.481 | 0.078 | **<0.001** | -0.450 | 0.078 | **<0.001** | -0.397 | | 0.078 | **<0.001** |
| UA5 | -0.517 | 0.084 | **<0.001** | -0.445 | 0.087 | **<0.001** | -0.344 | 0.086 | **<0.001** | -0.359 | | 0.085 | **<0.001** |
| PD2 | -0.138 | 0.074 | 0.062 | -0.044 | 0.075 | 0.557 | -0.190 | 0.075 | **0.011** | 0.033 | | 0.075 | 0.660 |
| PD3 | 0.049 | 0.066 | 0.452 | 0.049 | 0.069 | 0.473 | 0.139 | 0.069 | **0.044** | 0.175 | | 0.070 | **0.012** |
| PD4 | -0.670 | 0.079 | **<0.001** | -0.673 | 0.081 | **<0.001** | -0.661 | 0.081 | **<0.001** | -0.857 | | 0.082 | **<0.001** |
| PD5 | -0.327 | 0.081 | **<0.001** | -0.338 | 0.083 | **<0.001** | -0.344 | 0.083 | **<0.001** | -0.385 | | 0.081 | **<0.001** |
| AD2 | -0.060 | 0.074 | 0.421 | -0.203 | 0.078 | **0.009** | -0.144 | 0.077 | 0.062 | -0.079 | | 0.077 | 0.301 |
| AD3 | 0.032 | 0.072 | 0.659 | 0.078 | 0.074 | 0.292 | 0.129 | 0.074 | 0.083 | 0.246 | | 0.075 | **0.001** |
| AD4 | -0.340 | 0.079 | **<0.001** | -0.333 | 0.080 | **<0.001** | -0.367 | 0.080 | **<0.001** | -0.512 | | 0.081 | **<0.001** |
| AD5 | -0.339 | 0.076 | **<0.001** | -0.501 | 0.078 | **<0.001** | -0.391 | 0.078 | **<0.001** | -0.356 | | 0.077 | **<0.001** |
| Death | -2.573 | 0.126 | **<0.001** | -2.607 | 0.131 | **<0.001** | -2.654 | 0.130 | **<0.001** | -2.678 | | 0.128 | **<0.001** |
| Interaction terms | | | | | | | | | | | | | |
| MO2 | -0.017 | 0.084 | 0.843 | -0.022 | 0.087 | 0.803 | -0.037 | 0.087 | 0.669 | 0.040 | | 0.087 | 0.645 |
| MO3 | -0.074 | 0.077 | 0.341 | -0.033 | 0.079 | 0.678 | 0.061 | 0.077 | 0.434 | -0.060 | | 0.078 | 0.447 |
| MO4 | 0.053 | 0.111 | 0.634 | -0.082 | 0.113 | 0.471 | 0.180 | 0.113 | 0.109 | -0.041 | | 0.112 | 0.717 |
| MO5 | -0.028 | 0.163 | 0.864 | 0.198 | 0.167 | 0.238 | -0.117 | 0.170 | 0.492 | 0.201 | | 0.161 | 0.211 |
| SC2 | -0.100 | 0.103 | 0.332 | 0.002 | 0.107 | 0.988 | 0.015 | 0.108 | 0.887 | -0.085 | | 0.107 | 0.428 |
| SC3 | -0.018 | 0.098 | 0.851 | 0.004 | 0.100 | 0.968 | -0.098 | 0.101 | 0.331 | 0.072 | | 0.101 | 0.473 |
| SC4 | 0.125 | 0.101 | 0.214 | -0.008 | 0.104 | 0.938 | 0.090 | 0.103 | 0.384 | -0.067 | | 0.105 | 0.528 |
| SC5 | -0.037 | 0.120 | 0.759 | -0.005 | 0.124 | 0.969 | 0.051 | 0.123 | 0.676 | 0.175 | | 0.123 | 0.156 |
| UA2 | 0.126 | 0.103 | 0.222 | -0.026 | 0.108 | 0.810 | 0.087 | 0.108 | 0.419 | 0.067 | | 0.105 | 0.525 |
| UA3 | -0.015 | 0.091 | 0.873 | -0.041 | 0.094 | 0.660 | -0.068 | 0.093 | 0.468 | -0.028 | | 0.094 | 0.765 |
| UA4 | -0.037 | 0.105 | 0.726 | 0.035 | 0.110 | 0.752 | -0.073 | 0.110 | 0.509 | 0.013 | | 0.110 | 0.909 |
| UA5 | 0.008 | 0.118 | 0.947 | 0.116 | 0.123 | 0.345 | 0.108 | 0.122 | 0.378 | 0.077 | | 0.119 | 0.518 |
| PD2 | -0.016 | 0.104 | 0.881 | -0.079 | 0.106 | 0.453 | 0.008 | 0.105 | 0.940 | -0.238 | | 0.105 | **0.024** |
| PD3 | 0.049 | 0.092 | 0.595 | -0.121 | 0.097 | 0.211 | -0.126 | 0.097 | 0.191 | 0.002 | | 0.098 | 0.983 |
| PD4 | 0.066 | 0.111 | 0.551 | 0.138 | 0.115 | 0.228 | 0.059 | 0.114 | 0.605 | 0.125 | | 0.116 | 0.279 |
| PD5 | 0.013 | 0.115 | 0.907 | 0.050 | 0.118 | 0.668 | 0.115 | 0.116 | 0.322 | 0.086 | | 0.115 | 0.451 |
| AD2 | -0.026 | 0.105 | 0.804 | -0.182 | 0.110 | 0.099 | -0.083 | 0.109 | 0.448 | -0.001 | | 0.108 | 0.992 |
| AD3 | 0.090 | 0.101 | 0.374 | 0.028 | 0.104 | 0.792 | -0.054 | 0.104 | 0.604 | -0.138 | | 0.106 | 0.192 |
| AD4 | -0.045 | 0.111 | 0.682 | 0.013 | 0.113 | 0.911 | 0.049 | 0.113 | 0.667 | 0.145 | | 0.113 | 0.199 |
| AD5 | 0.056 | 0.107 | 0.600 | 0.122 | 0.110 | 0.266 | -0.024 | 0.109 | 0.829 | 0.019 | | 0.108 | 0.863 |
| Death | 0.483 | 0.177 | **0.006** | -0.043 | 0.185 | 0.816 | 0.022 | 0.184 | 0.906 | -0.043 | | 0.181 | 0.814 |
| LL |  |  | -10285 |  |  | -9604 |  |  | -9547 |  | |  | -9488 |
| AIC |  |  | 20654 |  |  | 19292 |  |  | 19177 |  | |  | 19061 |
| BIC |  |  | 20960 |  |  | 19595 |  |  | 19481 |  | |  | 19365 |

Note. Significant terms are bolded; Coef: coefficient estimate; SE: standard error; LL: log-likelihood; AIC: Akaike’s information criteria; BIC: Bayesian information criteria; Study arm 1, a 3-month life expectancy with limited support vignette; Study arm 2, a 3-month life expectancy with adequate support vignette; Study arm 3, a 2-year life expectancy with limited support vignette; Study arm 4, a 2-year life expectancy with adequate support vignette.

*Table 4b.* Separate MNLs with main effects and interactions between a dummy indicating a vignette with or without a context and each attribute level for each study arm in the UK sample

|  | Study Arm 1 | | | Study Arm 2 | | | Study Arm 3 | | | Study Arm 4 | | | | |
| --- | --- | --- | --- | --- | --- | --- | --- | --- | --- | --- | --- | --- | --- | --- |
|  | Coef. | SE | P. | Coef. | SE | P. | Coef. | SE | P. | Coef. | SE | P. |  |  |
| MO2 | -0.258 | 0.064 | **<0.001** | -0.289 | 0.063 | **<0.001** | -0.247 | 0.063 | **<0.001** | -0.169 | 0.064 | **0.009** |  |  |
| MO3 | -0.453 | 0.057 | **<0.001** | -0.456 | 0.055 | **<0.001** | -0.571 | 0.056 | **<0.001** | -0.412 | 0.056 | **<0.001** |  |  |
| MO4 | -0.019 | 0.083 | 0.818 | 0.160 | 0.080 | **0.046** | 0.046 | 0.079 | 0.561 | 0.227 | 0.082 | **0.006** |  |  |
| MO5 | -0.878 | 0.125 | **<0.001** | -1.066 | 0.123 | **<0.001** | -0.544 | 0.116 | **<0.001** | -1.150 | 0.123 | **<0.001** |  |  |
| SC2 | -0.141 | 0.079 | 0.074 | -0.153 | 0.077 | **0.046** | -0.206 | 0.076 | **0.006** | -0.320 | 0.079 | **<0.001** |  |  |
| SC3 | -0.033 | 0.074 | 0.655 | -0.026 | 0.073 | 0.724 | 0.075 | 0.072 | 0.302 | 0.033 | 0.074 | 0.649 |  |  |
| SC4 | -0.617 | 0.078 | **<0.001** | -0.596 | 0.076 | **<0.001** | -0.526 | 0.077 | **<0.001** | -0.648 | 0.077 | **<0.001** |  |  |
| SC5 | -0.138 | 0.093 | 0.139 | -0.169 | 0.090 | 0.061 | -0.301 | 0.090 | **0.001** | 0.007 | 0.092 | 0.937 |  |  |
| UA2 | -0.236 | 0.079 | **0.003** | -0.080 | 0.077 | 0.301 | -0.029 | 0.077 | 0.706 | -0.223 | 0.079 | **0.005** |  |  |
| UA3 | 0.275 | 0.071 | **<0.001** | 0.252 | 0.068 | **<0.001** | 0.173 | 0.068 | **0.010** | 0.198 | 0.070 | **0.005** |  |  |
| UA4 | -0.491 | 0.081 | **<0.001** | -0.567 | 0.080 | **<0.001** | -0.464 | 0.080 | **<0.001** | -0.416 | 0.082 | **<0.001** |  |  |
| UA5 | -0.355 | 0.088 | **<0.001** | -0.477 | 0.088 | **<0.001** | -0.413 | 0.086 | **<0.001** | -0.403 | 0.087 | **<0.001** |  |  |
| PD2 | -0.072 | 0.079 | 0.363 | -0.209 | 0.076 | **0.006** | -0.307 | 0.076 | **<0.001** | -0.074 | 0.079 | 0.344 |  |  |
| PD3 | 0.091 | 0.073 | 0.210 | 0.088 | 0.071 | 0.212 | 0.384 | 0.070 | **<0.001** | 0.098 | 0.073 | 0.178 |  |  |
| PD4 | -0.617 | 0.086 | **<0.001** | -0.687 | 0.083 | **<0.001** | -0.777 | 0.082 | **<0.001** | -0.693 | 0.084 | **<0.001** |  |  |
| PD5 | -0.341 | 0.084 | **<0.001** | -0.387 | 0.083 | **<0.001** | -0.222 | 0.082 | **0.007** | -0.509 | 0.084 | **<0.001** |  |  |
| AD2 | -0.198 | 0.079 | **0.012** | -0.305 | 0.078 | **<0.001** | -0.156 | 0.078 | **0.047** | -0.071 | 0.079 | 0.369 |  |  |
| AD3 | 0.185 | 0.078 | **0.018** | 0.262 | 0.077 | **0.001** | 0.158 | 0.077 | **0.041** | 0.098 | 0.078 | 0.208 |  |  |
| AD4 | -0.528 | 0.085 | **<0.001** | -0.621 | 0.083 | **<0.001** | -0.485 | 0.083 | **<0.001** | -0.497 | 0.084 | **<0.001** |  |  |
| AD5 | -0.261 | 0.081 | **0.001** | -0.332 | 0.078 | **<0.001** | -0.385 | 0.078 | **<0.001** | -0.306 | 0.080 | **<0.001** |  |  |
| Death | -3.024 | 0.136 | **<0.001** | -3.261 | 0.133 | **<0.001** | -2.962 | 0.133 | **<0.001** | -3.193 | 0.137 | **<0.001** |  |  |
| Interaction terms | | | | | | | | | | | | | |  |
| MO2 | 0.069 | 0.090 | 0.443 | -0.094 | 0.088 | 0.286 | -0.012 | 0.089 | 0.890 | -0.053 | 0.091 | 0.561 |  |  |
| MO3 | 0.026 | 0.081 | 0.751 | 0.023 | 0.078 | 0.767 | 0.068 | 0.079 | 0.383 | -0.008 | 0.079 | 0.924 |  |  |
| MO4 | 0.102 | 0.118 | 0.388 | -0.178 | 0.114 | 0.117 | 0.056 | 0.111 | 0.616 | -0.168 | 0.115 | 0.144 |  |  |
| MO5 | -0.074 | 0.177 | 0.676 | 0.429 | 0.172 | **0.013** | -0.130 | 0.164 | 0.428 | 0.301 | 0.171 | 0.079 |  |  |
| SC2 | 0.008 | 0.111 | 0.945 | -0.085 | 0.108 | 0.430 | -0.004 | 0.107 | 0.971 | 0.132 | 0.111 | 0.234 |  |  |
| SC3 | -0.102 | 0.103 | 0.326 | -0.048 | 0.102 | 0.639 | -0.116 | 0.101 | 0.250 | -0.132 | 0.103 | 0.202 |  |  |
| SC4 | 0.245 | 0.108 | **0.023** | 0.158 | 0.106 | 0.135 | 0.013 | 0.107 | 0.903 | 0.067 | 0.109 | 0.535 |  |  |
| SC5 | -0.131 | 0.131 | 0.317 | -0.031 | 0.126 | 0.804 | 0.108 | 0.127 | 0.396 | -0.208 | 0.130 | 0.110 |  |  |
| UA2 | -0.003 | 0.111 | 0.976 | -0.086 | 0.108 | 0.423 | -0.040 | 0.108 | 0.713 | 0.281 | 0.111 | **0.012** |  |  |
| UA3 | -0.046 | 0.099 | 0.643 | -0.047 | 0.095 | 0.621 | -0.017 | 0.095 | 0.861 | -0.069 | 0.099 | 0.483 |  |  |
| UA4 | 0.069 | 0.112 | 0.539 | 0.109 | 0.111 | 0.329 | 0.041 | 0.112 | 0.714 | -0.109 | 0.115 | 0.341 |  |  |
| UA5 | -0.076 | 0.124 | 0.538 | 0.049 | 0.123 | 0.687 | 0.022 | 0.122 | 0.859 | 0.073 | 0.122 | 0.552 |  |  |
| PD2 | 0.033 | 0.111 | 0.763 | 0.053 | 0.107 | 0.620 | 0.185 | 0.107 | 0.084 | -0.082 | 0.111 | 0.456 |  |  |
| PD3 | 0.009 | 0.101 | 0.928 | 0.065 | 0.099 | 0.511 | -0.102 | 0.099 | 0.302 | -0.059 | 0.102 | 0.563 |  |  |
| PD4 | -0.094 | 0.120 | 0.430 | 0.053 | 0.116 | 0.650 | 0.075 | 0.115 | 0.513 | 0.066 | 0.119 | 0.577 |  |  |
| PD5 | 0.005 | 0.119 | 0.967 | -0.006 | 0.117 | 0.958 | -0.117 | 0.116 | 0.312 | 0.022 | 0.119 | 0.851 |  |  |
| AD2 | 0.083 | 0.112 | 0.459 | 0.154 | 0.110 | 0.161 | 0.007 | 0.110 | 0.946 | -0.154 | 0.111 | 0.165 |  |  |
| AD3 | -0.080 | 0.109 | 0.460 | -0.091 | 0.107 | 0.391 | -0.069 | 0.109 | 0.527 | 0.237 | 0.110 | 0.031 |  |  |
| AD4 | 0.086 | 0.119 | 0.469 | 0.055 | 0.115 | 0.635 | 0.062 | 0.116 | 0.591 | -0.025 | 0.119 | 0.834 |  |  |
| AD5 | -0.162 | 0.114 | 0.156 | 0.033 | 0.110 | 0.764 | 0.099 | 0.109 | 0.364 | -0.093 | 0.113 | 0.410 |  |  |
| Death | 0.577 | 0.190 | **0.002** | 0.406 | 0.185 | **0.029** | 0.272 | 0.186 | 0.144 | 0.180 | 0.192 | 0.350 |  |  |
| LL |  |  | -8842 |  |  | -9288 |  |  | -9220 |  |  | -8616 |  |  |
| AIC |  |  | 17769 |  |  | 18659 |  |  | 18524 |  |  | 17317 |  |  |
| BIC |  |  | 18070 |  |  | 18963 |  |  | 18828 |  |  | 17619 |  |  |

Note. Significant terms are bolded; Coef: coefficient estimate; SE: standard error; LL: log-likelihood; AIC: Akaike’s information criteria; BIC: Bayesian information criteria; Study arm 1, a 3-month life expectancy with limited support vignette; Study arm 2, a 3-month life expectancy with adequate support vignette; Study arm 3, a 2-year life expectancy with limited support vignette; Study arm 4, a 2-year life expectancy with adequate support vignette.
